# MiR-30c/PGC-1β protects against diabetic cardiomyopathy via PPARα

**DOI:** 10.1186/s12933-019-0811-7

**Published:** 2019-01-11

**Authors:** Zhongwei Yin, Yanru Zhao, Mengying He, Huaping Li, Jiahui Fan, Xiang Nie, Mengwen Yan, Chen Chen, Dao Wen Wang

**Affiliations:** 1 Division of Cardiology and Hubei Key Laboratory of Genetics and Molecular Mechanisms of Cardiological Disorders, Tongji Hospital, Tongji Medical College, Huazhong University of Science and Technology, Wuhan, 430030 China; 2 Department of Cardiology, China-Japan Friendship Hospital, No. 2 Yinghua Dongjie, Beijing, 100029 China

**Keywords:** PGC-1β, miR-30c, Diabetic cardiomyopathy, Cardiac metabolism

## Abstract

**Background:**

Metabolic abnormalities have been implicated as a causal event in diabetic cardiomyopathy (DCM). However, the mechanisms underlying cardiac metabolic disorder in DCM were not fully understood.

**Results:**

Db/db mice, palmitate treated H9c2 cells and primary neonatal rat cardiomyocytes were employed in the current study. Microarray data analysis revealed that PGC-1β may play an important role in DCM. Downregulation of PGC-1β relieved palmitate induced cardiac metabolism shift to fatty acids use and relevant lipotoxicity in vitro. Bioinformatics coupled with biochemical validation was used to confirm that PGC-1β was one of the direct targets of miR-30c. Remarkably, overexpression of miR-30c by rAAV system improved glucose utilization, reduced excessive reactive oxygen species production and myocardial lipid accumulation, and subsequently attenuated cardiomyocyte apoptosis and cardiac dysfunction in db/db mice. Similar effects were also observed in cultured cells. More importantly, miR-30c overexpression as well as PGC-1β knockdown reduced the transcriptional activity of PPARα, and the effects of miR-30c on PPARα was almost abated by PGC-1β knockdown.

**Conclusions:**

Our data demonstrated a protective role of miR-30c in cardiac metabolism in diabetes via targeting PGC-1β, and suggested that modulation of PGC-1β by miR-30c may provide a therapeutic approach for DCM.

**Electronic supplementary material:**

The online version of this article (10.1186/s12933-019-0811-7) contains supplementary material, which is available to authorized users.

## Introduction

Diabetes mellitus is one of the major public health burdens in both developed and developing countries. The global prevalence of diabetes is predicted to be 4.4% in 2030 [1]. Diabetic patients frequently show cardiac abnormalities and even heart failure, which can be partly explained by conventional risk factors, such as hypertension and coronary artery disease (CHD) [2]. However, multiple epidemiological evidences revealed that there were still 2.4- to 5-fold increase of risk in developing heart failure between patients with diabetes and age/gender matched controls after conventional risk factors adjustment [3]. Recently, it was found that diabetes can lead to cardiomyocyte injury directly without endothelium dysfunction, named diabetic cardiomyopathy (DCM) [4]. DCM is defined as a unique form of abnormal ventricular structure and performance that occurs independently of hypertension, CHD or other potential cardiogenic etiology in diabetic patients, and its prevalence is estimated to be between 30 and 60% in preclinical and clinical stages among diabetic population [5, 6]. DCM is initially characterized by a hidden subclinical cardiac remodeling, and followed by noticeable diastolic dysfunction, later by systolic function damage, and eventually by clinical heart failure [7]. DCM has been confirmed as the leading cause of hospitalization and mortality in diabetic patients [5, 8]. Despite advances in molecular etiologies, current therapy approaches failed to prevent long-term cardiac events in patients with diabetes, which stressing the importance of novel therapeutic strategies.

Although there are multifactorial mechanisms involved in DCM, it is well accepted that cardiac metabolic derangement plays a central role in its occurrence and development [9–11]. With omnivorous and metabolically flexible characters, healthy adult hearts use energy from various substrates depending on the circumstances [12]. Physiologically, fatty acid (FA) contributes around 70% of the energy supply for working heart, whereas the remainder energy source primarily relies on glucose [12]. However, insulin resistance and hyperlipemia result in marked reduction in the ability to use glucose, and thus cardiomyocytes rely almost exclusively on FA derived energy [13, 14]. This substrate switch is accompanied by higher rates of oxygen consumption and impaired oxidative phosphorylation that result in reactive oxygen (ROS) overproduction. Concurrently, redundant FA is diverted to cellular neutral lipid pools or converted to toxic intermediates [15]. Subsequent injuries in mitochondria and other cell components was identified as the main triggers of cardiomyocyte apoptosis, death and consequential contractile dysfunction [10].

microRNAs (miRNAs) are a class of highly evolutionarily conserved endogenous single-stranded small (~ 22 nt) non-coding RNAs, which participate in numerous biological processes and diseases. miRNAs typically suppress gene expression at post-transcriptional levels by directly binding to the 3′ untranslated region (UTR) of target mRNAs [16]. Multiple miRNAs are identified to play key roles in the etiology of DCM [17, 18]. Our previous study also showed that miR-21 protected against diabetic cardiomyopathy induced diastolic dysfunction by targeting gelsolin [19]. miRNA based therapy for the prevention and treatment of DCM becomes promising in next-generation diabetic care [18].

In the present study, we found that PGC-1β was upregulated in the heart of db/db mice, and contributed to the cardiac metabolic disturbances. Then, we demonstrated that PGC-1β was regulated by miR-30c in vivo and in vitro. Most importantly, we showed that exogenous miR-30c delivery by recombinant adeno-associated virus system was sufficient to attenuate cardiac metabolic derangement and cardiac dysfunction in db/db mice. Mechanistically, we found that the effects of miR-30c/PGC-1β on DCM were, at least in part, mediated by PPARα signaling pathway.

## Research design and methods

### Microarray data, identification of DEGs and pathway enrichment analysis

Dataset GSE4745 (Affymetrix platform GPL85) was downloaded from Gene Expression Omnibus (https://www.ncbi.nlm.nih.gov/geo/query/acc.cgi?acc=GSE4745). GSE4745 contained global mRNA expression in heart tissues of control and streptozocin induced type I diabetic rats. The rats were sacrificed at 3 days (baseline), 28 days (diastolic dysfunction) and 42 days (both diastolic and systolic dysfunction) after streptozocin injection, respectively. The analyses were carried out using R project (v3.4.1) with packages from Bioconductor website.

The gene expression matrix was obtained from data of CEL format by background correction, quartile data normalization and annotation. The classical t-test was used to identify different expression genes (DEGs) with a cutoff p-value of 0.05.

KEGG is a knowledge base for systematic analysis of gene functions, linking genomic information with higher-order functional information [20]. KEGG pathway analyses were performed using DAVID bioinformatics resources and p < 0.05 was considered statistically significant [21].

### miRNA target prediction

Bioinformatics websites miRBase (http://www.mirbase.org) [22], TargetScan (http://www.targetscan.org) [23], PicTar (http://www.pictar.org) [24], DIANA (http://diana.imis.athena-innovation.gr/DianaTools) [25], and RNAhybrid (https://bibiserv.cebitec.uni-bielefeld.de/rnahybrid) [26] were used for predicting miRNAs that may target PGC-1β. Intersectional analyses of prediction results from these tools were performed using R project (v3.4.1).

### Reagents

Cell culture medium (DMEM and OPTI-MEM) and fetal bovine serum (FBS) were purchased from GIBCO (Thermo Fisher Scientific, Waltham, MA). The reverse transcriptional and real-time PCR primers of miR-30c and U6, mimics and inhibitor of miR-30c, siRNAs targeting PGC-1β and PPARα, and their negative controls were from RiboBio (Guangzhou, China). Lipofectamine 2000 and Trizol were from Invitrogen (Thermo Fisher Scientific, Waltham, MA). The primers of mRNA real-time PCR were designed and synthesized by BGI (Shenzhen, China). Antibodies against CD36, CPT1B, PDK4, GLUT4 and β-actin were from ABclonal Biotech (Cambridge, MA). Anti-Ago2 antibody was from Novus Biologicals (Littleton, CO). Antibodies against PGC-1β and PPARα were from Abcam (Cambridge, MA). Polyvinylidene difluoride membranes were from Millipore (Merck KGaA, Darmstadt, Germany). The horseradish peroxidase-conjugated secondary antibody and enhanced chemiluminescence reagents were from Pierce Biotech (Thermo Fisher Scientific, Waltham, MA). pTK-PPREx3-Luc was a kind gift from Dr. Bruce Spiegelman (Addgene plasmid Cat# 1015) [27]. Other reagents were from Sigma-Aldrich (Merck, St. Louis, MO) unless specified description elsewhere.

### Cell culture

H9c2 and HEK293T (American Type Culture Collection, Manassas, VA) were routinely cultured in DMEM medium supplemented with 10% FBS. H9c2 cells were transfected with miRNA mimics/inhibitor (100 nM), siRNAs (100 nM) with Lipofectamine 2000 according to manufacturer’s description, respectively, and then subjected to 1 mM palmitate (bound to BSA at a ratio of 5:1) stimulation for 48 h.

Primary cardiomyocytes were prepared from neonatal rats (day 0–3) and identified by immunofluorescent staining with antibody against cardiomyocyte-specific marker ACTN2 as previously described [28]. Primary cells were cultured with DMEM supplemented with 10% FBS and treated with 300 µM saturated FFA palmitate (16:0) for 48 h.

### Dual luciferase assays

Luciferase activity was detected using the Dual Luciferase Reporter Assay System (Promega, Beijing, China) as described previously [29].

To evaluate the binding of miRNA and target mRNA, the sequence containing predicted miR-30c binding site of the human PGC-1β 3′ UTR and corresponding mutant sequence (Additional file 1: Table S1) were synthesized and inserted into pMIR-REPORT luciferase vector (Ambion, Thermo Fisher Scientific, Waltham, MA). To determine whether miR-30c targeting 3′ UTR of PGC-1β, HEK293T cells were co-transfected with appropriate pMIR construct (1 μg/mL), pRL-TK plasmid (0.1 μg/mL) with miR-30c mimics or negative controls (100 nM).

To evaluate the effects of PGC-1β or miR-30c on PPARα transcriptional activity, H9c2 cells were first transfected with PGC-1β siRNA or miR-30c, and then transfected with pTK-PPREx3-Luc plasmid (1 μg/mL), respectively [30]. To determine whether miR-30c regulated PPARα transcriptional activity via PGC-1β, H9c2 cells were transfected with miR-30c and then transfected pTK-PPREx3-Luc plasmid (1 μg/mL) after PGC-1β inhibition by siRNA.

Transfections were done in duplicates and repeated at least three times in independent experiments.

### Preparation of rAAV system and animal study

All animal procedures were approved by the Institutional Animal Care and Use Committee of Tongji Medical College of Huazhong University of Science and Technology (HUST), and complied with ARRIVE and the *Guide for the Care and Use of Laboratory Animals* standards (NIH publication).

The recombinant adeno-associated virus of type-9 (rAAV9) system was a kind gift from Dr. Xiao Xiao (University of North Carolina at Chapel Hill). Synthesized fragments of miR-30c, anti-miR-30c, or miR-random (Additional file 1: Table S2) were inserted into rAAV expression plasmid, respectively. The rAAVs were prepared by triple plasmid co-transfection in human HEK293T cells as described previously [29].

Male db/db mice with C57BL/Ks background and their control littermates were bred in Model Animal Research Center of Nanjing University and maintained in Laboratory Animal Center of HUST. Db/db mice were randomly assigned to four groups (n = 8–10 for each group). A single tail vein injection of the rAAV-miR-random, rAAV-miR-30c, or rAAV-anti-miR-30c (1 × 10^11^ vector genomes in 200 μL PBS per mouse) [31] was performed at the age of 8 weeks in db/db mice, whereas db/db control and C57BL/Ks mice were treated with equal volume of PBS in the same way. At the age of 28 weeks, echocardiographic and hemodynamic analyses were performed and then mice were sacrificed with anaesthetization by the mixture of xylazine and ketamine. Tissue samples were taken for further study.

### Echocardiographic and hemodynamic measurements

Echocardiographic measurements were performed under anesthesia with 1.5% isoflurane using a high-resolution imaging system with a 30-MHz high-frequency scanhead (Vevo770, VisualSonics Inc, Toronto, Canada) as previously described [29]. Hemodynamic measurements of left ventricle were performed under anesthesia with intraperitoneal injection of the mixture of ketamine and xylazine using a Millar Catheter System (Millar 1.4F, SPR 835, Millar Instruments Inc, Houston, TX) as described previously [29].

### Western blots analyses

Western blotting was performed using specific antibodies as described previously [29]. The band intensities of target proteins were analyzed by using ImageJ program [29].

### ChIP–PCR

Chromatin immunoprecipitation (ChIP) assays were conducted using a kit from Beyotime (Nanjing, China), following the manufacturer’s protocol [32]. Primers used for ChIP–PCR were listed in Additional file 1: Table S3.

### Real-time PCR

Total RNA was isolated from heart tissues and H9c2 cells with Trizol reagent, and was reverse-transcribed into cDNA using RevertAid First Strand cDNA Synthesis Kit (Thermo Fisher Scientific, Waltham, MA). Real-time PCR assays were performed using SYBR rapid quantitative PCR Kit (Kapa Biosystems, Woburn, MA) [33]. The relative miRNA levels were determined by normalizing to U6 level.

To assess the mitochondrial biogenesis, the ratio of mitochondrial to genomic DNA was used. Real-time PCR was carried out with different primers (Additional file 1: Table S4). 18S ribosomal DNA was used to represent genomic DNA; 18S ribosomal DNA and COI was used to represent mitochondrial DNA.

### Electron microscopy

Fresh specimen of mice hearts was fixed with glutaraldehyde in PBS, dehydrated, coated and then observed by electron microscopy analysis (FEI Tecnai G2, Hillsboro, OR) [33]. Lipid droplets were counted in ten images from different fields.

### TUNEL assay

To determine cell apoptosis in myocardial tissue sections, TdT-mediated dUTP nick-end labeling (TUNEL) assay was performed using a kit of R&D Systems (Minneapolis, MN) according to the manufacturer’s instructions [33]. Pictures of slides were obtained by using a microscope (Nikon, Tokyo, Japan) equipped with digital imaging camera.

### Staining of heart samples

Neutral lipids accumulating in cultured H9c2 cells was stained with BODIPY 493/503 (Invitrogen, Thermo Fisher Scientific, Waltham, MA) and then imaged by the fluorescence microscopy (Nikon, Tokyo, Japan). Myocardial lipids accumulation in mouse heart frozen sections was stained by oil Red O and then imaged by the ordinary optics microscopy as previously described [32].

To determine intracellular ROS production in myocardial tissue sections, dihydroethidium (DHE, Beyotime, Nanjing, China) staining was performed according to the manufacturer’s instructions [29]. Pictures of slides were obtained by using a fluorescence microscopy microscope (Nikon, Tokyo, Japan) equipped with digital imaging camera.

### Flow cytometry analyses

To evaluate cell apoptosis, H9c2 cells were incubated with Annexin V-FITC/PI (BD Biosciences, Sparks, MD) [33]. To evaluate intracellular ROS production, H9c2 cells were incubated with DCFH-DA (Beyotime, Nanjing, China) [29]. After incubation, cells were analyzed with a FACStar Plus flow cytometer (BD Biosciences, Sparks, MD) as previously described [8].

### ATP level determination

The ATP level was determined using an Enhanced ATP Assay Kit (Beyotime, Nanjing, China) according to the manufacturer’s instructions. Briefly, cells were lysed with ATP lysis buffer on ice and centrifuged. The supernatant was then transferred to the ATP working solution and mixed quickly. A luminometer (Promega, Beijing, China) was used to measure the luminescence immediately. The luminescence data were normalized by the total protein levels of individual samples.

### Detection of mitochondrial membrane potential

The mitochondrial membrane potential (ΔΨm) was assessed using a JC-1 kit from Invitrogen (Thermo Fisher Scientific, Waltham, MA) according to manufacturer’s description.

### Statistical analyses

All data were shown as mean ± SEM unless otherwise noted. Differences between groups were evaluated using Student’s t-test of unpaired data or one-way analysis of variance (ANOVA) and Bonferroni post-test. p < 0.05 was considered statistically significant. All statistical tests were performed using GraphPad Prism (v5.0) and SPSS (v18.0) unless otherwise stated.

## Results

### PGC-1β was increased in diabetic heart

**Fig. 1:**
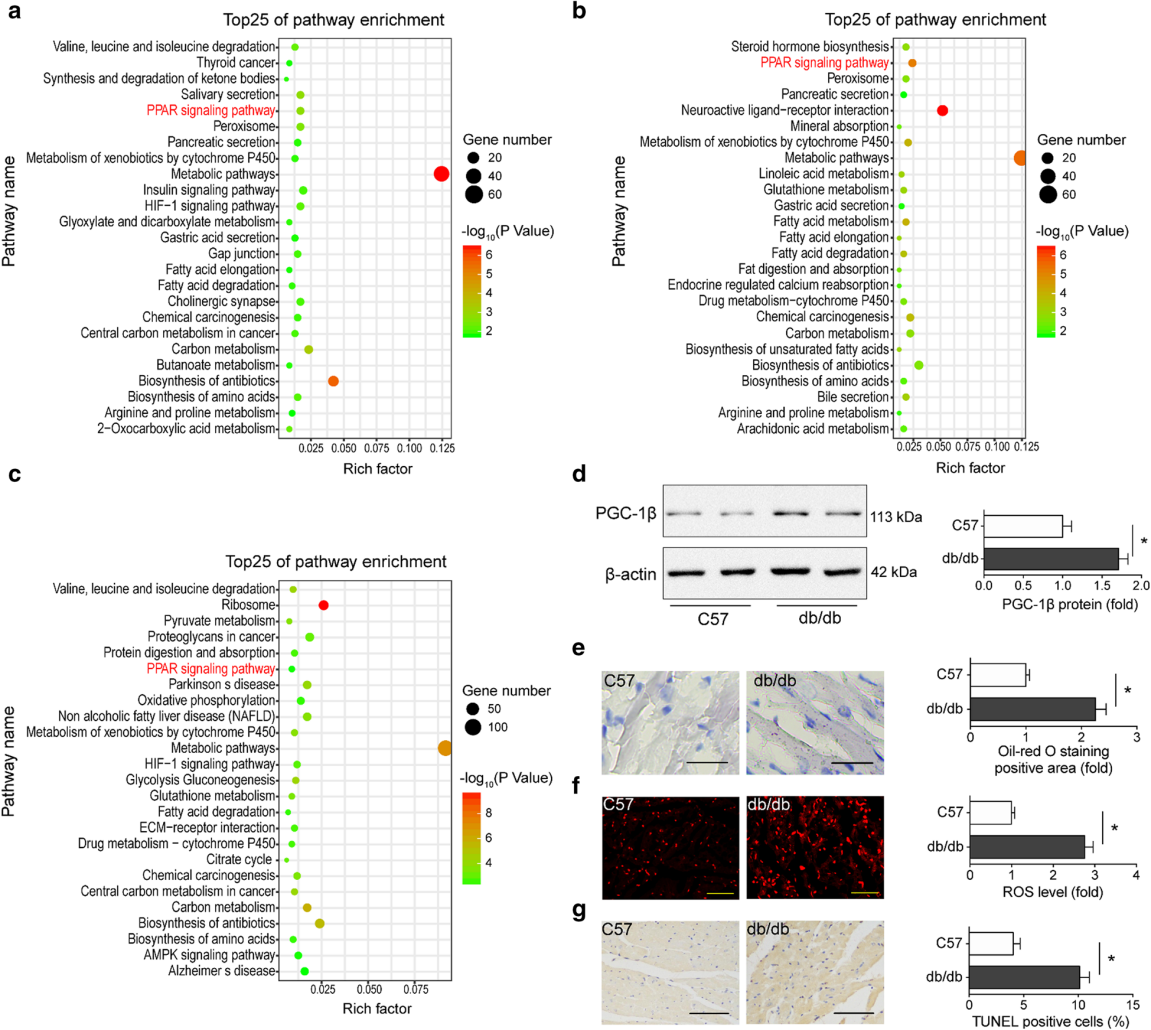
PGC-1β was increased in diabetic heart. **a**–**c** KEGG pathway analysis of DEGs between diabetic hearts and normal ones. Genes altered at 3 day (**a**), 28 day (**b**), 42 day (**c**) after diabetes induction were obtained by statistical reanalysis of the GSE4745 data. Bubble diagrams were generated using ggplot2 package. The y-axis shows the name of KEGG pathway; and the enrichment factor in x-axis denotes the ratio of the number of DEGs to the number of all unigenes in each reference pathway. The color of the dot represents p-value, and the size of the dot represents the exact number of DEGs mapped to this pathway. Cardiac tissues used in **d**–**g** were from 28-week-old male db/db mice and their C57 littermates. **d** Western blots and quantitative analysis of PGC-1β in the heart tissues. β-actin was used as an internal control. **e** Representative images and quantitative analysis of oil red O staining of cardiac lipid deposition (n = 5–8, bar = 50 μm). **f** Representative images and quantification of DHE staining of cardiac ROS production (n = 5–8, bar = 50 μm). **g** Representative images and quantification of TUNEL assay determining cell apoptosis in heart tissues (n = 5–8, bar = 100 μm). For **d**–**f**, data are expressed as mean ± SEM, *p < 0.05. DEG indicates differently expressed gene. *DHE* dihydroethidium, *ROS* reactive oxygen species, *TUNEL* TdT-mediated dUTP nick-end labeling

We re-analyzed the GSE4745 dataset, which contained mRNA expression profiles of in hearts from normal and diabetic rats. According to the finding criterion p value < 0.05, a total of 331, 370 and 865 differentially expressed genes (DEGs) were identified during the progression of DCM, respectively (Additional file 2). By analyzing the KEGG pathway of DEGs using DAVID bioinformatics resources, we found that hearts from diabetic rats exhibit a global disorder of metabolism relative pathways. We noticed that PPAR pathway was among the top enrichment pathways consistently (Fig. 1a–c). Specifically, FA transport and oxidation associated genes were significantly altered in PPAR pathway (Additional file 1: Figure S1A–C). PGC-1β is an important coactivator of PPAR, but its roles in diabetic heart have not yet been elucidated. Therefore, we focused on PGC-1β related cardiac substrate metabolism in further study.

To explore the role of PGC-1β in DCM, its expression in heart tissues of db/db mice were measured by Western blot at the age of 28 weeks, when both the cardiac diastolic and systolic function was impaired. The results showed that cardiac PGC-1β was significantly increased in db/db mice compared with C57 littermates (Fig. 1d). On the other hand, we detected substantially increased triglycerides accumulation, ROS level and cell apoptosis in heart sections of db/db mice in comparison with controls, indicating abnormal FA transport and oxidation in diabetic heart (Fig. 1e–g). These data suggested that PGC-1β may play important roles in DCM.

### PGC-1β knockdown relieved high palmitate induced lipotoxicity in vitro

**Fig. 2:**
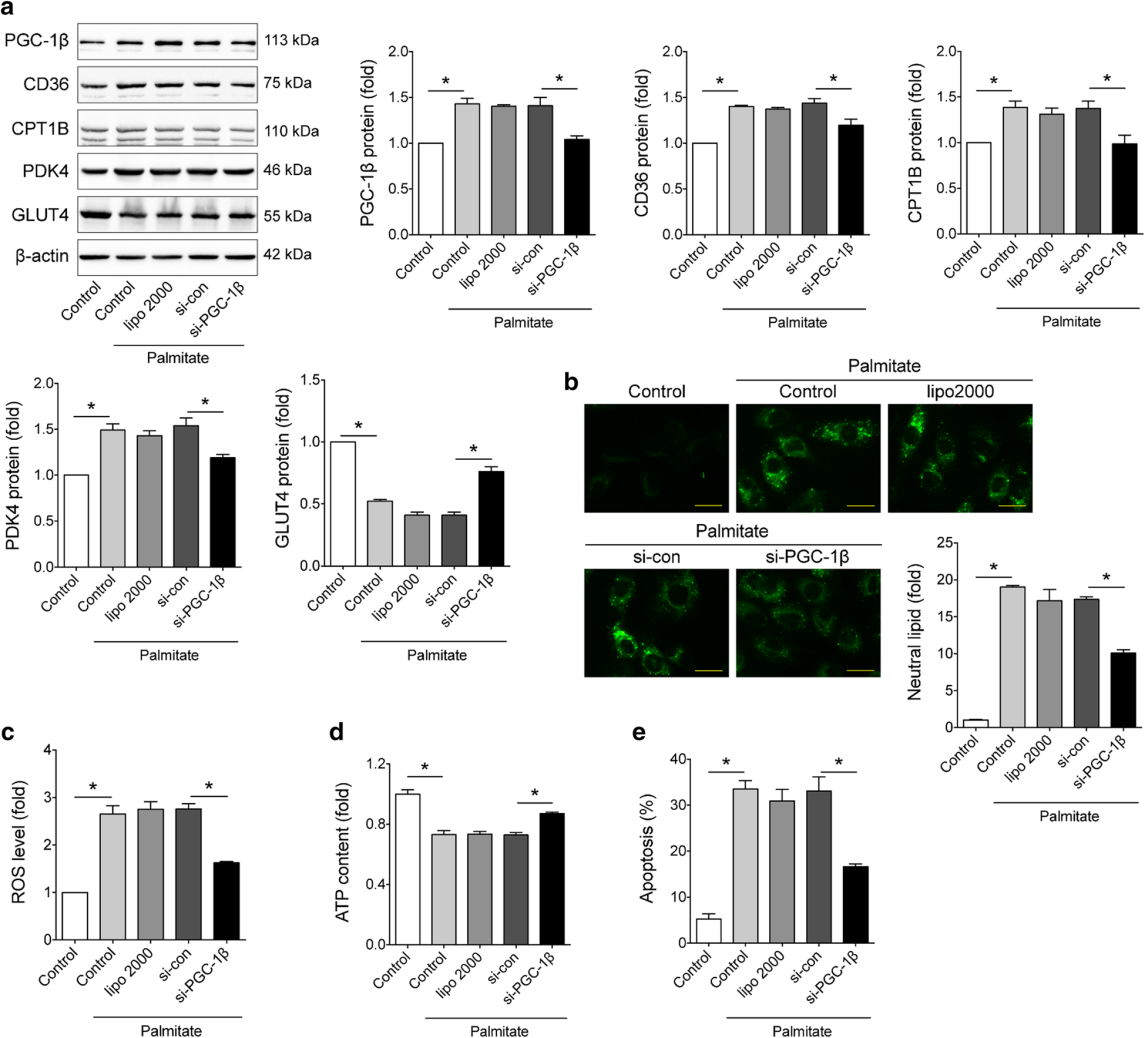
PGC-1β knockdown relieved high palmitate induced lipotoxicity in vitro. H9c2 cells were transfected with PGC-1β siRNA and then subjected to palmitate (1 mmol/L) stimulation. **a** Representative Western blots and quantification of PGC-1β, CD36, CPT1B, PDK4, GLUT4 in H9c2 cells with different treatment. β-actin was used as an internal control. **b** Representative images and quantitative analysis of BODIBY 493/503 fluorescent dye staining of neutral lipid level in cells (bar = 25 μm). **c** ROS generation in cells was examined by flow cytometry with DHE probe treatment. **d** ATP content of H9c2 cells with different treatment. **e** Apoptosis of H9c2 cells was measured by flow cytometry with Annexin V-PE and propidium iodide staining. For all panels, data are representative of three independent experiments and expressed as mean ± SEM, *p < 0.05

As FA transport and oxidation associated genes were significantly altered in diabetic heart (Fig. 1a–c), we then employed an in vitro model of lipotoxicity by incubating H9c2 cells in high-palmitate medium. Insulin signaling was markedly impaired after high-palmitate treatment (Additional file 1: Figure S2). Western blots showed that high-palmitate significantly increased the expression of PGC-1β (Fig. 2a). Three pairs of siRNAs were designed to knockdown PGC-1β expression, and the efficacy of the siRNAs was shown in Additional file 1: Figure S3. Metabolic disturbances of diabetic hearts are classically driven by abnormal expression of relative genes which increase FA uptake and utilization, such as CD36 and CPT1B, as well as decrease glucose metabolism, such as GLUT4 and PDK4 [34]. Consistently, high-palmitate treatment led to glucose use shift to fatty acids, evidenced by downregulation of GLUT4 and upregulation of PDK4, CD36 and CPT1B (Fig. 2a). Moreover, BODIPY staining manifested that high-palmitate treatment induced lipid accumulation in cardiomyocytes (Fig. 2b). Consistently, elevated ROS generation (Fig. 2c) and decreased ATP production (Fig. 2d) were significantly induced by high-palmitate treatment, indicating mitochondria damage. Importantly, the percentages of apoptotic cells were significantly higher in cells treated with high-palmitate than control (Fig. 2e). While all the effects induced by high-palmitate treatment were significantly alleviated upon PGC-1β knockdown (Fig. 2a–e).

Since H9c2 cells are undifferentiated myoblasts, neonatal rat cardiomyocytes (NRCMs) were then prepared (Additional file 1: Figure S4). Experiments in NRCMs also showed similar results (Additional file 1: Figure S5). In addition, considering PGC-1β is a key regulator of mitochondria, we additionally examined mitochondrial function and biogenesis. The results showed high-palmitate treatment significantly disrupted mitochondria membrane potential but induced mitochondrial biogenesis, while PGC-1β knockdown reduced the effects of high-palmitate (Additional file 1: Figure S6). Overall, these data suggested that PGC-1β may play crucial roles in DCM via lipotoxicity.

### PGC-1β expression was reduced by miR-30c

**Fig. 3:**
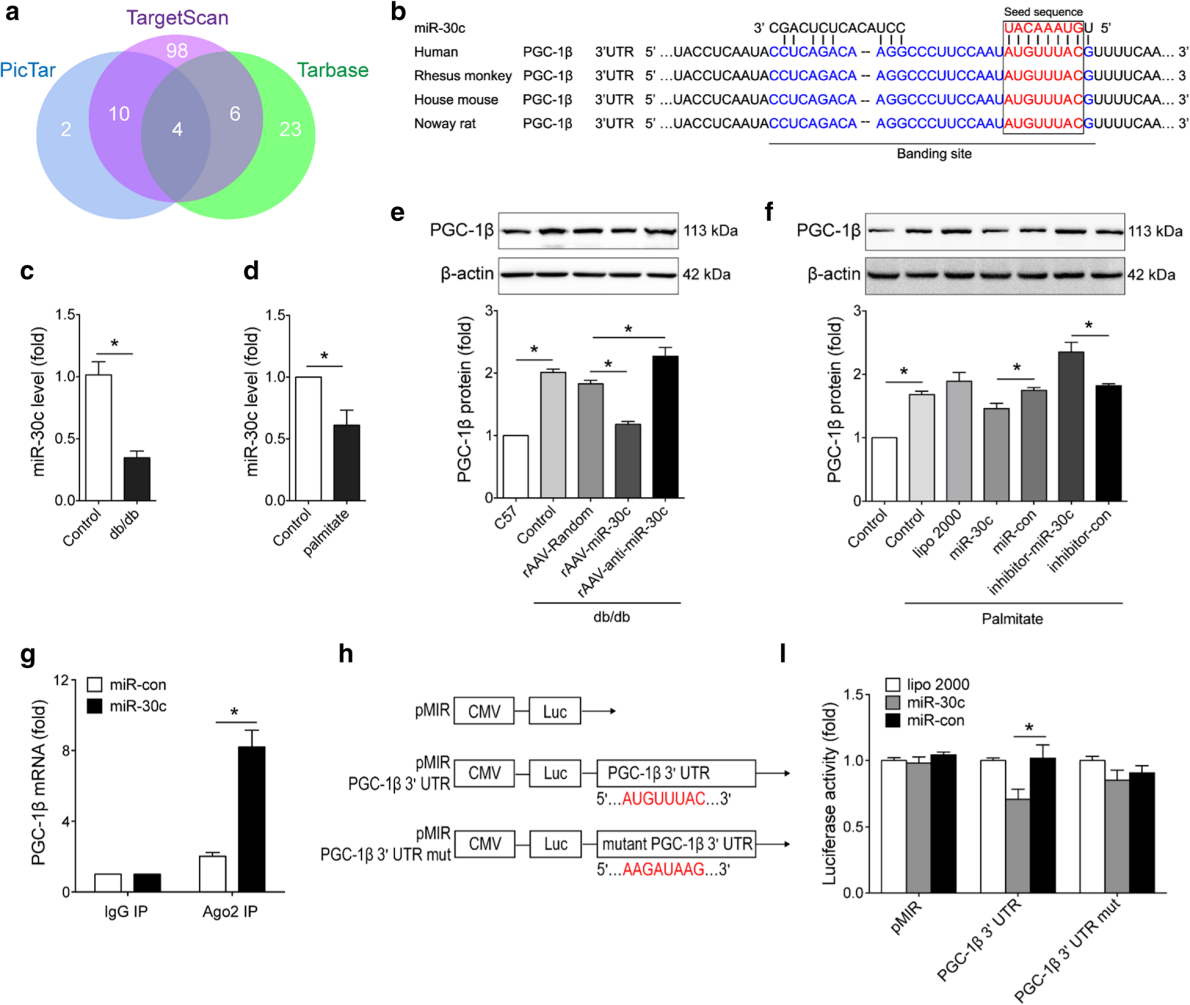
PGC-1β expression was reduced by miR-30c. **a** Venn diagram showing the overlap number of microRNA targeting PGC-1β predicted by TargetScan, PicTar and TarBase websites. **b** Sequence alignment of miR-30c on the 3′ UTR of PGC-1β from different organisms. **c** miR-30c expression was determined by RT-PCR in the heart tissues from 28-week old male db/db diabetic mice and their C57 littermates (n = 3). **d** miR-30c expression was determined by RT-PCR in H9c2 cells with or without palmitate (1 mmol/L) treatment. **e** Animals (db/db mice and C57 control mice) were injected with the corresponding rAAVs at 8–10 weeks of age and sacrificed at 28 weeks of age (n = 8–10). **f** H9c2 cells were transfected with miR-30c mimics/inhibitors (or their control) and then subjected to palmitate (1 mmol/L) stimulation. Representative Western blots and quantitative analysis of PGC-1β in the cardiac tissues (**e**) and H9c2 cells (**f**). β-actin was used as an internal control. **g** PGC-1β mRNA level was determined by RT-PCR in the whole RNA and the RNA of the anti-ago2 co-IP complex from H9c2 cells treated with miR-30c mimics or miR-control. **h** Schematic diagram of luciferase reporter plasmids pMIR PGC-1β 3′ UTR and mutant PGC-1β 3′ UTR, and the potential (or mutant) seed sequence of miR-30c targeting PGC-1β 3′ UTR are shown in red. **i** Regulation of PGC-1β via miR-30c targeting its 3′ UTR was determined with luciferase reporter assays in HEK293T cells. For **d**, **f**, **g** and **i**, n = three independent experiments. Data are expressed as mean ± SEM, *p < 0.05. *UTR* untranslated region, *RT-PCR* real-time polymerase chain reaction, *rAAV* recombinant adeno-associated virus, *IP* immunoprecipitation

To identify the potential miRNAs which may target PGC-1β, we screened the online computational tools PicTar, TargetScan and TarBase (Additional file 3: Table S2). Intersectional analyses of prediction results from these tools were shown in the Venn diagram (Fig. 3a). The combined overlap resulted in 4 candidate miRNAs—miR-30a, miR-30b, miR-30c and miR-30e (Additional file 3: Table S2). Previous studies from our lab and other groups have shown that miR-30c possessed a much higher abundance in H9c2 cardiomyocytes and heart tissues from C57 mice than other miR-30 s [33, 35, 36]. Moreover, multiple sequences alignment revealed that the potential binding sites and flank sequences between miR-30c and PGC-1β were highly conserved among human, rhesus monkey and rodents (Fig. 3b). Thus, we selected miR-30c in further study. Consistently, we found that cardiac miR-30c was significantly downregulated in db/db mice compared with C57 control (Fig. 3c). Meanwhile, the level of miR-30c was reduced in high-palmitate treated H9c2 cells compared with untreated control (Fig. 3d).

We next tested the effects of miR-30c on PGC-1β expression. Western blots showed that cardiac PGC-1β level was reduced in rAAV9-miR-30c-treated group compared with rAAV-Random-treated group, while rAAV9-anti-miR-30c treatment increased PGC-1β expression level in the heart (Fig. 3e). Consistently, miR-30c mimic transfection significantly reduced PGC-1β level in comparison with miR-con, while miR-30c inhibitor showed opposite effect (Fig. 3f).

Moreover, we performed RNA co-immunoprecipitation with anti-Ago2 and luciferase reporter assays. We found that Ago2 showed increased co-immunoprecipitation with the PGC-1β transcript after miR-30c transfection (Fig. 3g), which is consistent with the traditional machinery of miRNA-induced silencing complex. To verify a direct binding site of miR-30c on PGC-1β mRNA 3′ UTR, we cloned the wild-type or mutated sequence of PGC-1β 3′ UTR into pMIR vectors (Fig. 3h). When co-transfected with miR-30c mimics, the luciferase activity of PGC-1β 3′ UTR reporter but not its mutated form was significantly repressed compared with the transfection of miR-con (Fig. 3i). Together, these data indicated that miR-30c directly inhibited PGC-1β expression through targeting its 3′ UTR.

### Overexpression of miR-30c alleviated high palmitate induced lipotoxicity in vitro

**Fig. 4:**
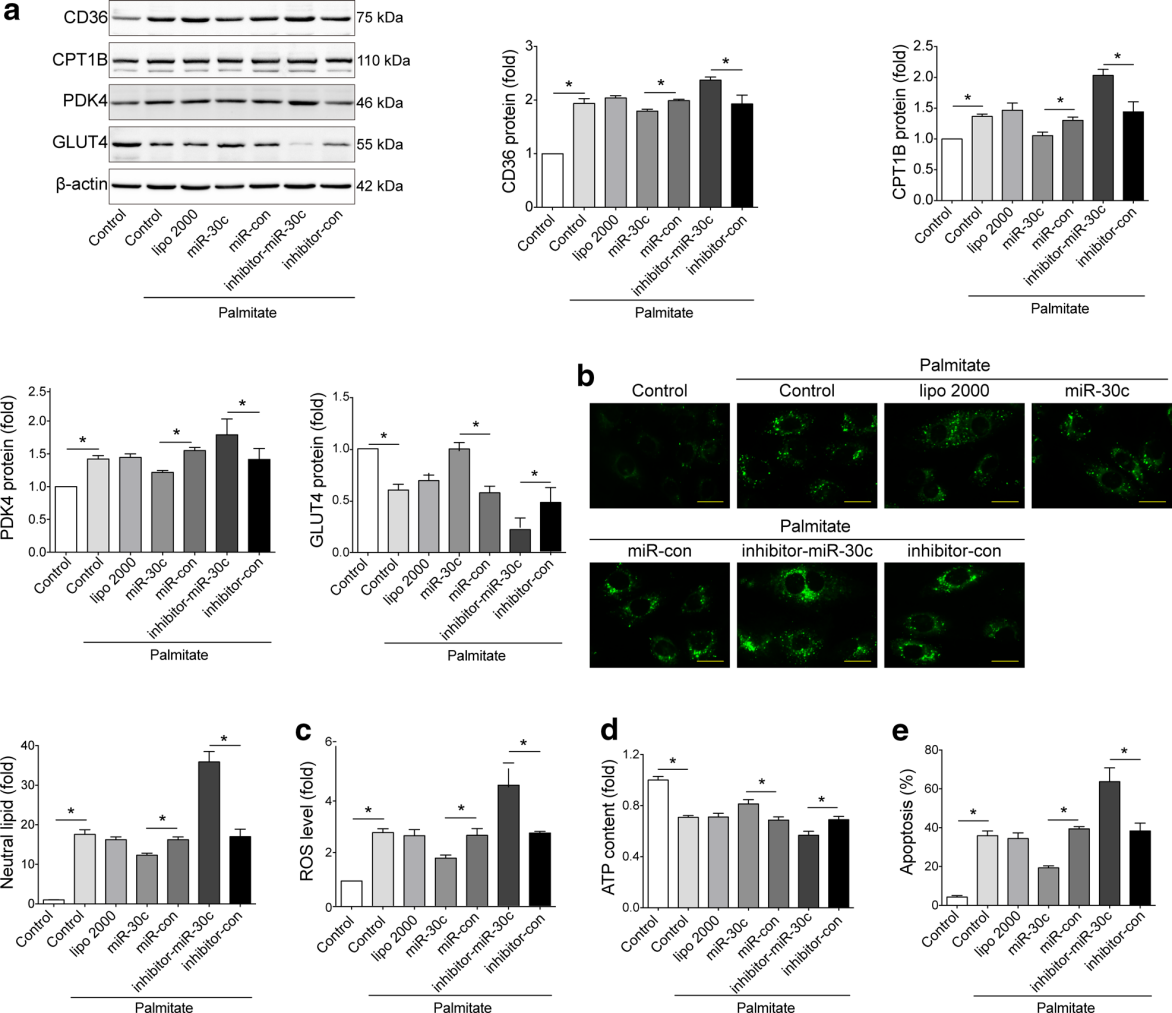
Overexpression of miR-30c alleviated high palmitate induced lipotoxicity in vitro. H9c2 cells were transfected with miR-30c mimics/inhibitors (or their control) and then subjected to palmitate (1 mmol/L) stimulation. **a** Representative Western blots and quantification of PGC-1β, CD36, CPT1B, PDK4, GLUT4 in H9c2 cells with different treatment. β-actin used as an internal control. **b** Representative images and quantitative analysis of BODIBY 493/503 fluorescent dye staining of neutral lipid level in cells (bar = 25 μm). **c** ROS generation in cells was examined by flow cytometry with DHE probe treatment. **d** ATP content of H9c2 cells with different treatment. **e** Apoptosis of H9c2 cells was measured by flow cytometry with Annexin V-PE and propidium iodide staining. For all panels, data are representative of three independent experiments and expressed as mean ± SEM, *p < 0.05

To investigate the effects of miR-30c/PGC-1β signaling, gain/loss-of-function analyses were conducted in H9c2 cells by transfecting miR-30c mimics/inhibitor followed by high-palmitate treatment. Similar to the siRNA against PGC-1β, miR-30c mimics reversed the high-palmitate treatment induced glucose-FA use shift compared with miR-con; while miR-30c inhibitor further enhanced the effect of high-palmitate (Fig. 4a). As expected, miR-30c mimics protected H9c2 cells from lipotoxicity, indicated by less lipid accumulation (Fig. 4b), decreased ROS level (Fig. 4c), improved ATP production (Fig. 4d), and reduced apoptosis (Fig. 4e) in comparison with si-con. While miR-30c inhibitor promoted lipotoxicity (Fig. 4b–e). These findings were further confirmed in experiments performed with NRCMs (Additional file 1: Figure S7). Also, miR-30c mimics significantly preserved mitochondria membrane potential and suppressed mitochondrial biogenesis in high-palmitate treated NRCMs (Additional file 1: Figure S8), while miR-30c inhibitor showed opposite effects. These results suggested that miR-30c protected H9c2 cells from hyperactive FA metabolism and lipotoxicity in vitro.

### Overexpression of miR-30c attenuated cardiac dysfunction in db/db mice

**Fig. 5:**
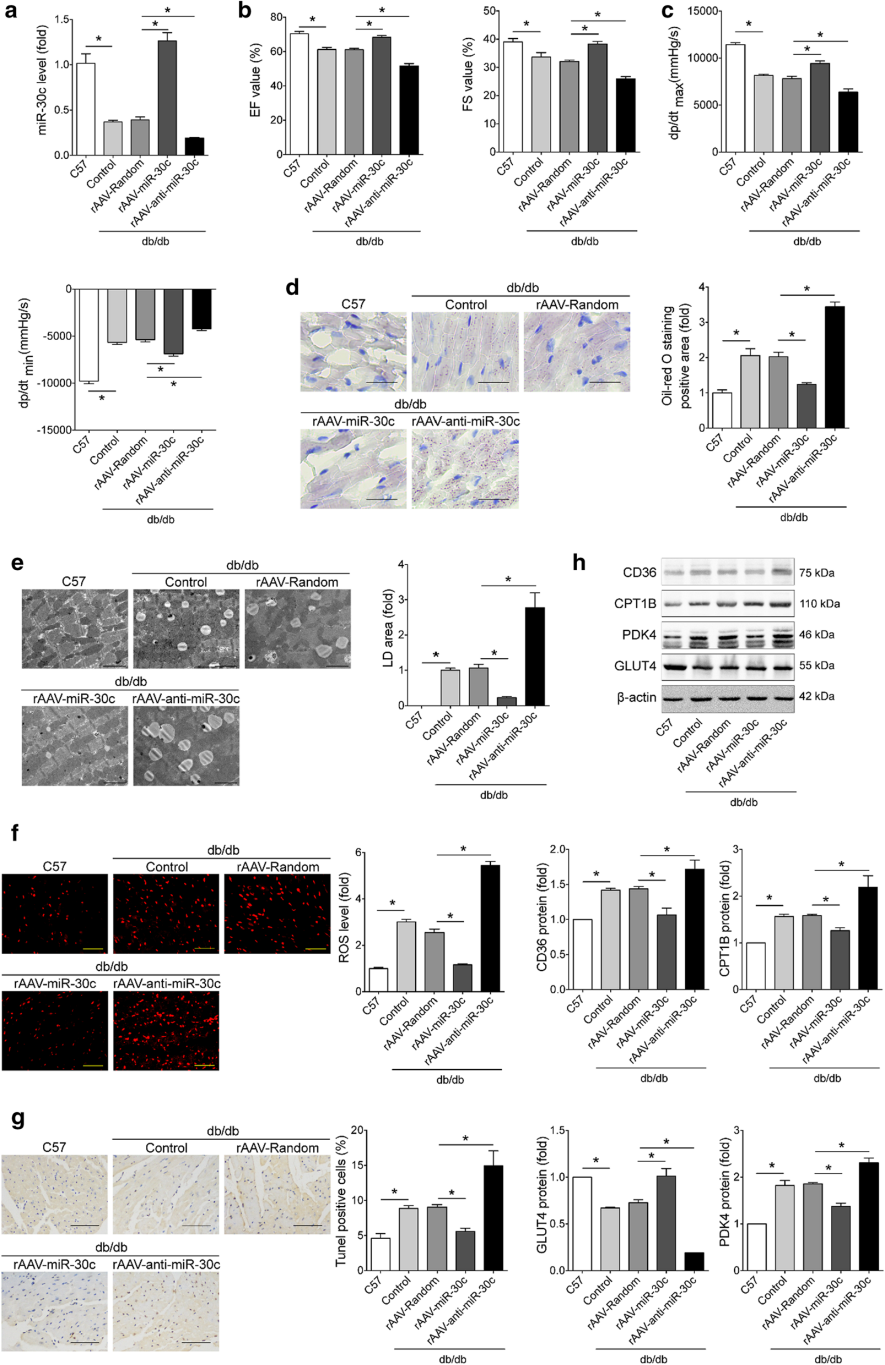
Overexpression of miR-30c attenuated cardiac dysfunction in db/db mice. Db/db mice and C57 control mice were injected with the corresponding rAAVs at 8–10 weeks of age and then sacrificed at 28 weeks of age (n = 8–10). **a** miR-30c expression was determined by RT-PCR in the heart tissues of mice. **b** Echocardiographic detection of mice. **c** Hemodynamic analysis measured by Millar cardiac catheter system of mice. **d** Representative images and quantitative analysis of oil red O staining of cardiac lipid deposition (bar = 50 μm). **e** Representative images and quantitative analysis of lipid droplets from cardiac tissues analyzed by transmission electron microscopy (bar = 2 μm). **f** Representative images and quantification of DHE staining of cardiac ROS production (bar = 50 μm). **g** Representative images and quantification of TUNEL assay determining cell apoptosis in heart tissues (bar = 100 μm). H. Representative Western blots and quantification of CD36, CPT1B, PDK4, GLUT4 in heart tissues with different treatment. β-actin used as an internal control. Data are expressed as mean ± SEM, *p < 0.05. *EF* ejection fraction, *FS* fractional shortening, *dP/dt*_*max*_ peak instantaneous rate of left ventricular pressure increase, *dP/dt*_*min*_ peak instantaneous rate of decline in left ventricular pressure increase, *LD* lipid droplet

To further confirm that the role of miR-30c/PGC-1β signaling in vivo, rAAV9 system (1 × 10^11^ vector copy numbers) were employed to express miR-30c or anti-miR-30c in db/db mice. As shown in Fig. 5a, rAAV9-miR-30c increased miR-30c expression in the heart of db/db mice compared with rAAV9-Random, while rAAV9-anti-miR-30c decreased the expression of miR-30c. Echocardiographic and hemodynamic measurements showed that miR-30c overexpression efficiently relieved the cardiac dysfunction of db/db mice, but miR-30c inhibition exacerbated these dysfunction (Fig. 5b, c and Additional file 1: Table S5). Oil red O staining revealed that rAAV9-miR-30c treatment significantly decreased lipid deposition in db/db mice compared with rAAV9-Random treatment, while rAAV9-anti-miR-30c treatment aggravated lipid accumulation (Fig. 5d). Consistently, markedly fewer and smaller lipid droplets in cardiomyocytes were observed by transmission electron microscopy in miR-30c overexpressing db/db mice than in rAAV9-Random treated db/db mice, conversely, miR-30c knockdown increased the number and area of lipid droplets (Fig. 5e). Furthermore, DHE and TUNEL staining showed that overexpression of miR-30c significantly reduced, whereas miR-30c inhibition robustly augmented, ROS generation and cell apoptosis in hearts of db/db mice (Fig. 5f, g). In addition, miR-30c expression by rAAV9 reserved glucose-FA use shift in diabetic heart, in contrast, anti-miR-30c showed opposite effects (Fig. 5h). Thus, overexpression of miR-30c attenuated cardiac dysfunction via redressing the myocardial metabolic disturbance.

We also found rAAV-miR-30c significantly reduced levels of plasma triglyceride (TG) and LDL-C in db/db mice; while rAAV-anti-miR-30c exhibited the opposite effects (Additional file 1: Figure S9A, B). However, there was no significant difference in levels of plasma total cholesterol (TC), HDL-C, FFAs and blood glucose among db/db mice with different treatments (Additional file 1: Figure S9C–F). In addition, rAAV-miR-30c treatment relieved hepatic steatosis in db/db mice, while rAAV-ant-miR-30c aggravated steatosis in liver of db/db mice (Additional file 1: Figure S10).

### PGC-1β knockdown blocked PPARα transcriptional activity in diabetic heart

**Fig. 6:**
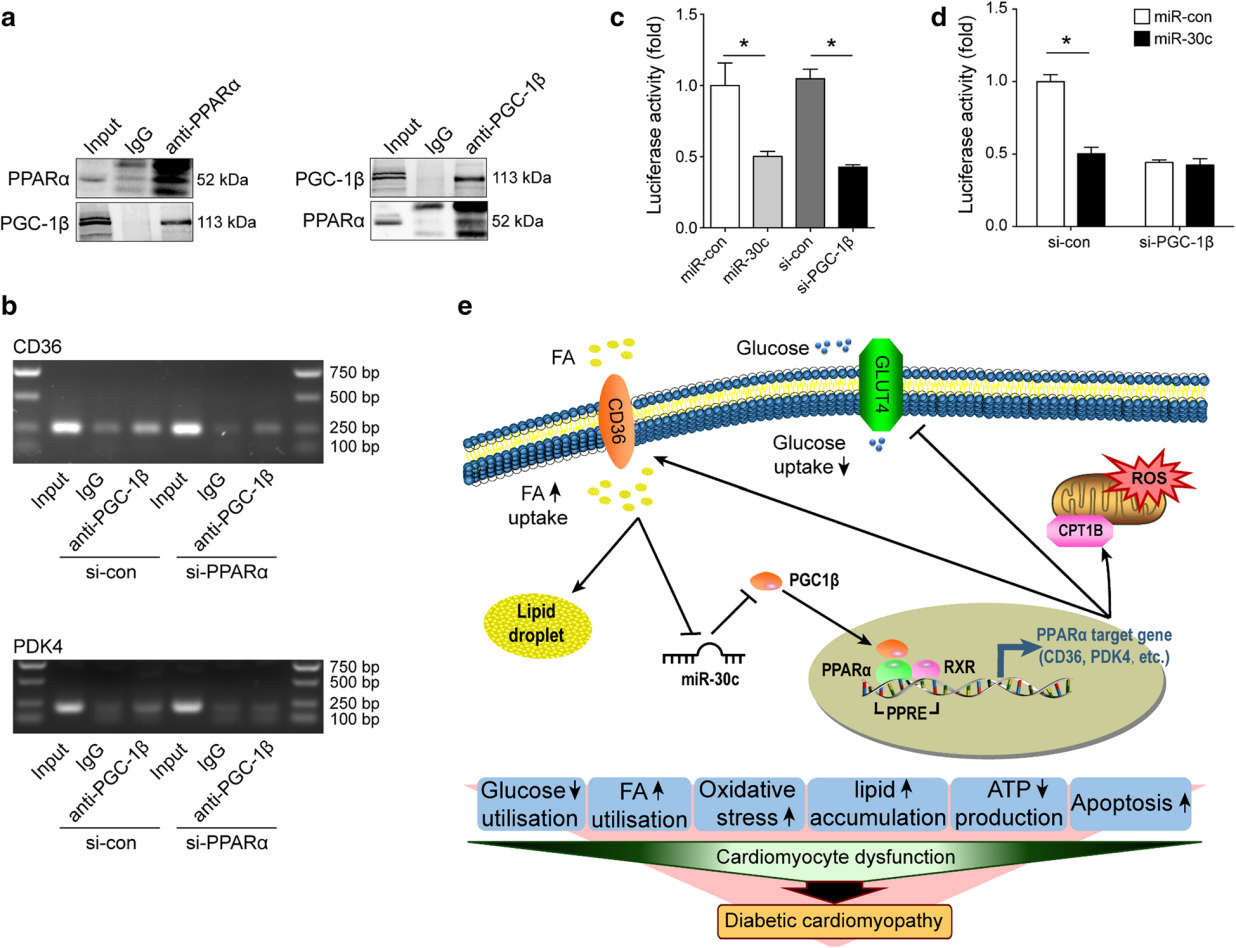
PGC-1β knockdown blocked PPARα transcriptional activity in diabetic heart. **a** Immunoprecipitation with the anti-PPARα (left) or anti-PGC-1β antibody (right) in H9c2 cells, followed by Western blotting with antibodies for anti-PPARα and anti-PGC-1β. **b** ChIP PCR for analysis of PGC-1β binding activity on the CD36 and PDK4 promoter in H9c2 cells treated with PPARα siRNA or siRNA control. Primers were designed according to PPARα/RXR binding site. **c** Effect of PGC-1β or miR-30c on PPARα transcriptional activity was evaluated by luciferase reporter assays in H9c2 cells. Cells were first transfected with PGC-1β or miR-30c, and then transfected with pTK-PPREx3-Luc plasmid. **d** Effect of miR-30c on PPARα transcriptional activity after PGC-1β inhibition was determined by luciferase reporter assays in H9c2 cells. Cells were transfected with miR-30c after PGC-1β inhibition by siRNA, and then transfected pTK-PPREx3-Luc plasmid. **e** Schematic representation of the association among miR-30c, PGC1β, and abnormality in diabetic heart. In cardiomyocyte of diabetic heart, miR-30c was decreased due to high fatty acid (FA). The loss of miR-30c resulted in PGC1β activation, which enhanced the PPARα transcriptional activity. Upregulated PPARα target genes leaded to glucose-FA use shift, oxidative stress, lipid accumulation, ATP production abnormality and apoptosis, which contributed to the pathologic process of diabetic cardiomyopathy. For **c**, **d**, data are representative of three independent experiments and expressed as mean ± SEM, *p < 0.05. *ChIP* chromatin immunoprecipitation assay, *PPRE* peroxisome proliferator response element

The above results prompted us to explore how miR-30c/PGC-1β signaling attenuate myocardial metabolic disorder in diabetic heart. As shown in Additional file 1: Figure S1, PPAR mediated FA transport and oxidation pathways were significantly enriched during the progression of DCM. Given PPARα is the most powerful isoform of PPARs in regulating FA transport and oxidation, we mainly focused on the interaction between PGC-1β and PPARα. Firstly, we verified the binding of PGC-1β and PPARα in H9c2 cells by co-immunoprecipitation assays (Fig. 6a). Then, we analyzed the promoter regions of PPARα target genes to find out whether PGC-1β was recruited to the PPARα binding site of these genes. CD36 and PDK4 were chosen as the representatives of PPARα target genes, because CD36 was an important gene involved in FA metabolism and PDK4 was the most critical gene associated with glucose utilization. As shown in Fig. 6b, the region between -798 and -568 of CD36 gene, and the region between − 1671 and − 1476 of PDK4 gene were enriched in ChIP-PCR assays, respectively, while this effect was abolished by pretreatment with PPARα siRNA (Fig. 6b, c). These results suggested that PGC-1β was recruited to promoters of these two genes via PPARα. Finally, pTK-PPREx3-Luc plasmid was transfected into H9c2 cells to evaluate the transcriptional activity of PPARα. As expected, miR-30c as well as si-PGC-1β pretreatment significantly reduced the reporter activity compared with miR-con or si-con, respectively, verifying the effects of miR-30c and PGC-1β on the PPARα transcriptional activity (Fig. 6d). However, PGC-1β knockdown completely blocked the reporter activity reduction induced by exogenous miR-30c treatment (Fig. 6d). These results indicated that miR-30c regulated PPARα transcriptional activity via PGC-1β.

## Discussion

Our present study found that miR-30c/PGC-1β/PPARα pathway involved in DCM via regulating cardiac metabolism. In the diabetic heart, the expression of miR-30c was repressed. The loss of miR-30c directly led to increased PGC-1β, which enhanced the transcriptional activity of PPARα. The upregulation of PPARα and its target genes in turn contributed to metabolic abnormalities and lipotoxicity in the heart, including enhanced FA uptake and oxidation, reduced glucose utilization, impaired ATP production, and subsequently cardiomyocyte apoptosis and cardiac dysfunction. Moreover, excessive FA uptake in turn repressed miR-30c expression, further promoted the progress of DCM. The protective roles of miR-30c/PGC-1β/PPARα in DCM via regulating cardiac metabolism were summarized in Fig. 6e.

There are indeed several different pathological manifestations of cardiac dysfunction between patients with type 1 and type 2 diabetes. For example, hyperglycemia-induced myocardial fibrosis may be predominant in type 1 diabetes, while cardiomyocyte hypertrophy is more related to the insulin resistance and dyslipidemia of type 1 diabetes [37]. However, multiple recent studies have shown that the underlying mechanisms and phenotypes of DCM in both types of diabetes mostly overlapped, especially for the cardiac metabolic abnormalities. Both types of diabetes shared similar downstream of a switch to greater cardiomyocyte FA utilization at the expense of glucose [38]. In animal models, both the STZ-induced and db/db cardiac dysfunction models were characterized by increased fatty acid utilization, decreased glucose utilization, impaired cardiac efficiency and function, compromised mitochondrial energetics, and increased lipid accumulation in the heart [10]. Moreover, different animal models of type 1 and type 2 diabetes have been used interchangeably in multiple studies to understand the pathophysiological mechanisms of DCM [39]. Therefore, we analyzed the expression dataset from STZ-induced models, because only these profiles covered three pathological stages of DCM, which may involve different molecular signals. Considering that type 2 diabetes was by far the most common type of diabetes, we investigated db/db mice model in the current study.

Multiple mechanisms are involved in the metabolic abnormalities of the diabetic heart, among which PPAR pathway was considered as an important one [11]. Previous study showed that mice with cardiac-restricted overexpression of PPARα (MHC-PPARα) exhibited a cardiac metabolic phenotype strikingly similar to diabetic heart, which was evidenced by increased FA utilization, decreased glucose utilization, myocyte lipid accumulation and cardiac dysfunction [40]. Whereas, excessive FA metabolism in diabetic heart was significantly relieved in PPARα knockout mice [41]. Previously, other group, as well as ourselves, have showed that inhibition of PPARα could alleviate atherogenic dyslipidemia and DCM [42, 43]. Consistently, our study identified that FA transport and oxidation associated genes were enriched in PPAR pathway in all three pathological stages of DCM via bioinformatics analysis.

We next investigated the key drivers of PPAR pathway and turned to PPAR co-activator PGC-1β. PGC-1β, together with PGC-1α, was identified as a master regulator of fatty acid oxidation gene expression in stress induced cardiac hypertrophy and heart failure [44]. Although the effects of PGC-1β on metabolic relative cardiac dysfunction, especially DCM, are hardly known, the upregulation of PGC-1β in diabetic heart has been observed in several animal models [32, 45]. PGC-1β was further determined as the top hub gene together with PPARα by transcriptome bioinformatics analysis in DCM [45]. In addition, PGC-1α has been widely accepted as the key contributor to the metabolic disorder in DCM via coactivating PPAR [10, 46]. PGC-1β shares similar tissue distribution pattern and significant sequence homology with PGC-1α [47]. In line with these findings, our study revealed that cardiac PGC-1β was significantly increased in DCM, and downregulation of PGC-1β protected cardiomyocytes from glucose-FA use shift and lipotoxicity.

PGC-1β have recently been discovered as powerful regulators of cellular metabolism by interacting with several different transcription factors including estrogen-related receptors (ERRs), nuclear respiratory factors (NRFs), and the PPAR family [47]. Our current study found that elevated PGC-1β promoted metabolic abnormalities via positively regulating PPARα transcriptional activity. However, whether other transcriptional factors, such as PPARγ and ERRγ, are involved in PGC-1β induced metabolic disorder in the diabetic heart need further study.

Most importantly, we have provided multiple lines of evidences demonstrating that PGC-1β is a target of miR-30c. Overexpression of miR-30c by rAAV system improved glucose utilization, reduced excessive ROS production and myocardial lipid accumulation, and subsequently attenuated cardiomyocyte apoptosis and cardiac dysfunction in vivo and in vitro. Our data revealed the protective roles of miR-30c in cardiac metabolism in diabetes via targeting PGC-1β. Previous studies found that both miR-30c and PGC-1β were involved in ER stress [48, 49]. Meanwhile, many studies have shown that excessive ER stress, trigger by hyperglycemia, free fatty acids (FFAs) and inflammation, played important roles in the development of DCM [50]. These evidences indicated that miR-30c/PGC1β may participate in the progression of DCM via ER stress.

We also found that rAAV9 mediated miR-30c delivery partly relieved hyperlipidemia and hepatic steatosis in db/db mice. Indeed, overexpression of miR-30c in liver has been demonstrated to decrease hepatic lipid biosynthesis and lipoprotein [35]. It is possible that the systemic effects of miR-30c contributed to its protective role in heart in our mice model. In addition, cardiac-restricted overexpression of PPARα developed DCM and fatty livers, indicating that cardiac metabolic remodeling could in turn induce systemic metabolic disorders [51]. In our current study, overexpression of miR-30c significantly reduced PPARα activity in heart of db/db mice, which might contribute to the remission of concurrent hyperlipidemia and hepatic steatosis.

Additionally, PGC-1β knockdown, as well as miR-30c overexpression, inhibited mitochondrial biogenesis but preserved mitochondrial function and ATP production in NRCMs treated with palmitate. Actually, increased mitochondrial biogenesis together with mitochondrial dysfunction has been reported as a characteristic of DCM [52]. Mitochondrial biogenesis may be a potentially adaptive mechanism in the face of compromised mitochondrial function and cardiac energetics. Since PGC-1β is a major regulator of mitochondrial biogenesis [53], it is possible that miR-30c/PGC-1β is involved in regulating this process. However, this has not been established definitively and need further study.

Taken together, our data demonstrated the protective role of miR-30c in cardiac metabolism in diabetes via targeting PGC-1β, and suggested that modulation of PGC-1β by miR-30c may provide a therapeutic approach for DCM.

## Conclusion

Taken together, we found that exogenous miR-30c delivered by rAAV system in db/db mice was sufficient to improve lipid and glucose utilization, reduce excessive ROS production and myocardial lipid accumulation, and subsequently attenuate cardiomyocyte apoptosis and cardiac dysfunction via PGC-1β/PPARα signals. Our study provided a new promising strategy for DCM.

## Additional files

**Additional file 1: Figure S1.** Different expression genes belonging to PPAR family between diabetic hearts and normal ones. **Figure S2.** High palmitate treatment impaired the insulin signal of NRCMs. **Figure S3.** The efficacy of siRNAs against PGC-1β. **Figure S4.** Identification of the primary neonatal rat cardiomyocytes. **Figure S5.** PGC-1β knockdown relieved high palmitate induced lipotoxicity in vitro. **Figure S6.** The effects of PGC-1β knockdown on mitochondrial biogenesis and membrane potentials. **Figure S7.** Overexpression of miR-30c alleviated high palmitate induced lipotoxicity in vitro. **Figure S8.** The effect of miR-30c on mitochondrial biogenesis and membrane potentials. **Figure S9.** The effects of rAAV9 mediated miR-30c/anti-miR-30c delivery on plasma lipid profile and blood glucose in vivo. **Figure S10.** The effects of rAAV9 mediated miR-30c/anti-miR-30c delivery on liver steatosis in vivo. **Table S1.** The wildtype sequence containing predicted miR-30c binding site of the human PGC-1β 3′ UTR and corresponding mutant sequence. **Table S2.** Sequences of miR-30c, anti-miR-30c, or miR-random inserted into rAAV expression plasmid. **Table S3.** Primers of CD36 and PDK4 promotors in ChIP-PCR. **Table S4.** Primers of rat 12S ribosomal DNA, COI and and 18S ribosomal DNA in real-time PCR. **Table S5.** Comparison of hemodynamic variables among mice with different treatments.

**Additional file 2.** **Table S1.** Differentially expressed genes in 3, 28 and 42 day after diabetes induction.

**Additional file 3.** **Table S2.** microRNA targeting PGC-1β predicted by TargetScan, PicTar and TarBase websites.

## Abbreviations

DCM: diabetic cardiomyopathy
CHD: coronary artery diseas
PPARα: peroxisome proliferators-activated receptor α
PGC-1β: peroxisome proliferator-activated receptor γ coactivator 1β
miRNA: microRNA
DEG: different expression gene
PCR: polymerase chain reaction
rAAV: recombinant adeno-associated virus
KEGG: kyoto encyclopedia of genes and genomes
PPRE: peroxisome proliferator response element
SEM: standard error of mean
ATP: adenosine triphosphate
FA: fatty acid
ROS: reactive oxygen species
UTR: untranslated region
FBS: fetal bovine serum
ChIP: chromatin immunoprecipitation
TUNEL: TdT-mediated dUTP nick-end labeling
ANOVA: one-way analysis of variance
ERRs: estrogen-related receptors
NRFs: nuclear respiratory factors
