# Supplementary material for: MiR-30c/PGC-1β protects against diabetic cardiomyopathy via PPARα
Source: Cardiovasc Diabetol. 2019 Jan 11;18:7. doi: 10.1186/s12933-019-0811-7 (PMC6329097; doi:10.1186/s12933-019-0811-7)
Supplement: Supplementary file 1 — Additional file 1: Figure S1. Different expression genes belonging to PPAR family between diabetic hearts and normal ones. Figure S2. High palmitate treatment impaired the insulin signal of NRCMs. Figure S3. The efficacy of siRNAs against PGC-1β. Figure S4. Identification of the primary neonatal rat cardiomyocytes. Figure S5. PGC-1β knockdown relieved high palmitate induced lipotoxicity in vitro. Figure S6. The effects of PGC-1β knockdown on mitochondrial biogenesis and membrane potentials. Figure S7. Overexpression of miR-30c alleviated high palmitate induced lipotoxicity in vitro. Figure S8. The effect of miR-30c on mitochondrial biogenesis and membrane potentials. Figure S9. The effects of rAAV9 mediated miR-30c/anti-miR-30c delivery on plasma lipid profile and blood glucose in vivo. Figure S10. The effects of rAAV9 mediated miR-30c/anti-miR-30c delivery on liver steatosis in vivo. Table S1. The wildtype sequence containing predicted miR-30c binding site of the human PGC-1β 3′ UTR and corresponding mutant sequence. Table S2. Sequences of miR-30c, anti-miR-30c, or miR-random inserted into rAAV expression plasmid. Table S3. Primers of CD36 and PDK4 promotors in ChIP-PCR. Table S4. Primers of rat 12S ribosomal DNA, COI and and 18S ribosomal DNA in real-time PCR. Table S5. Comparison of hemodynamic variables among mice with different treatments. [file 12933_2019_811_MOESM1_ESM.docx]

**Figure S1**

A


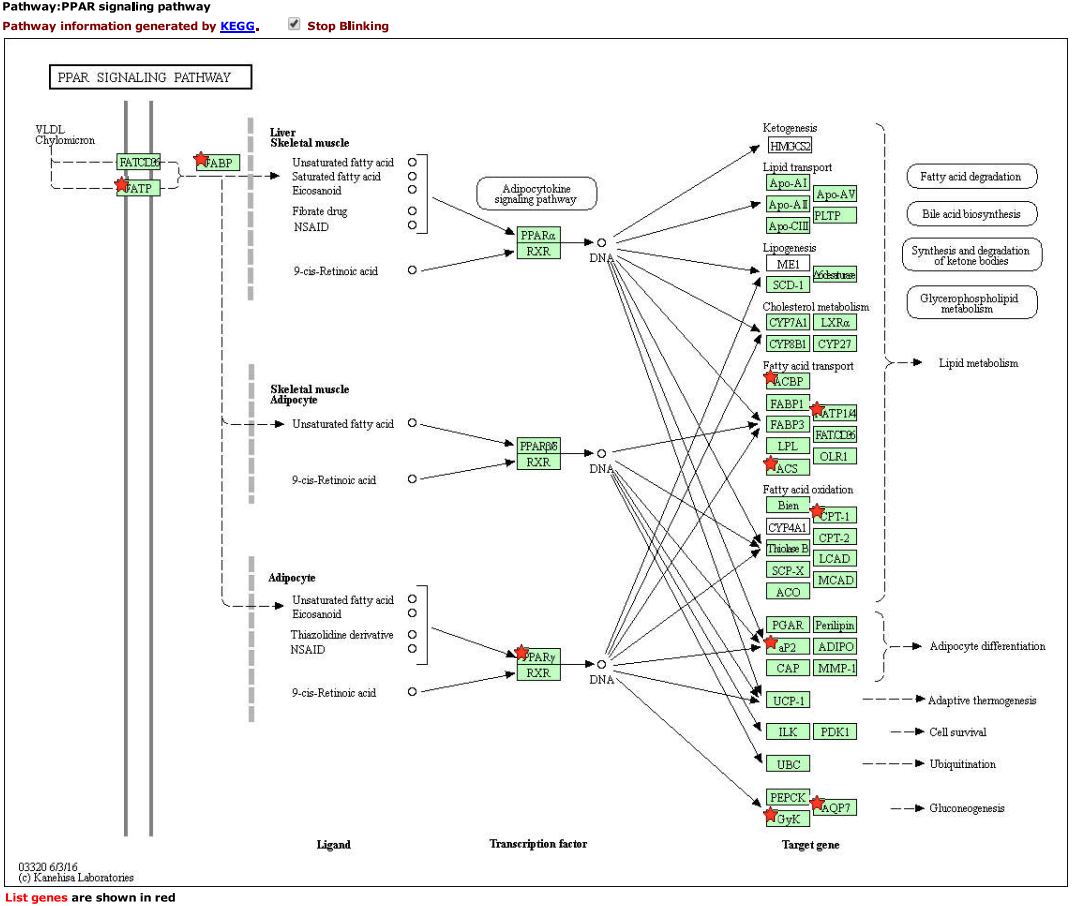


B


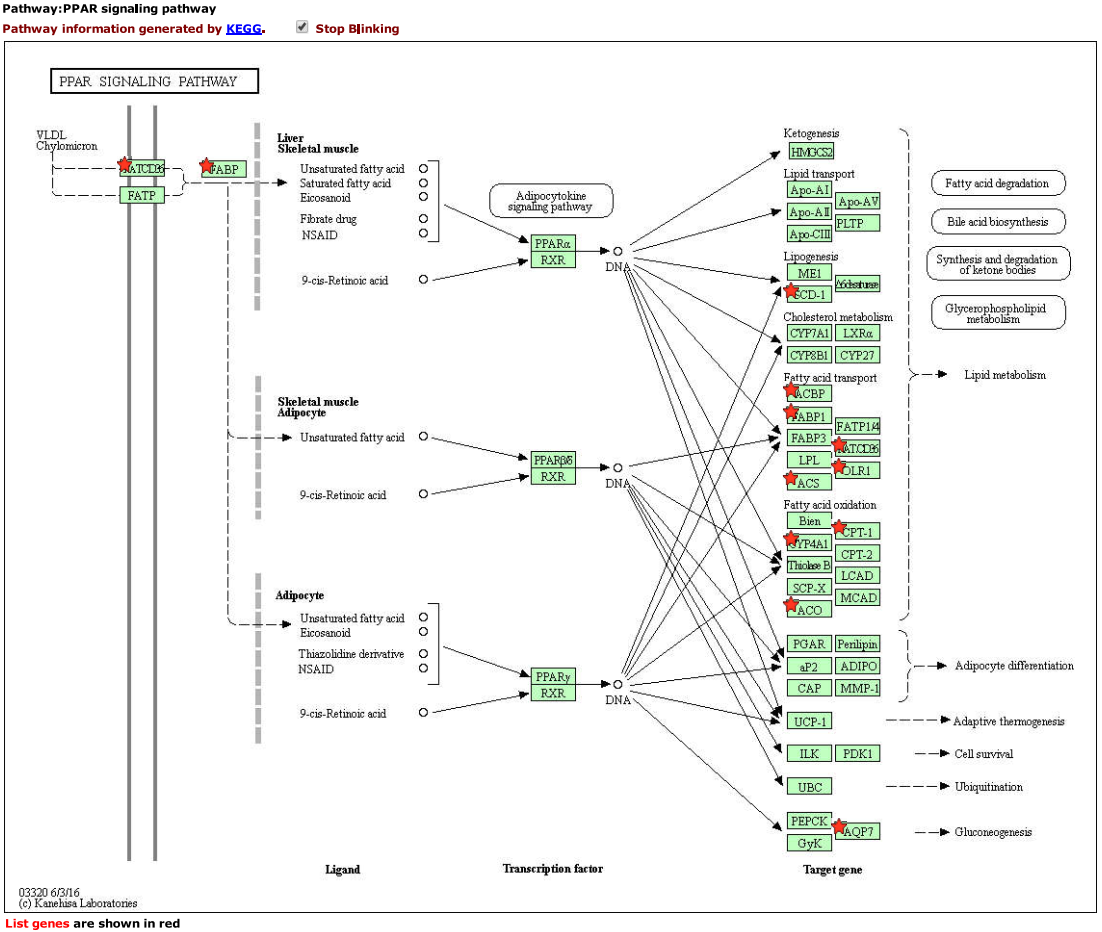


C


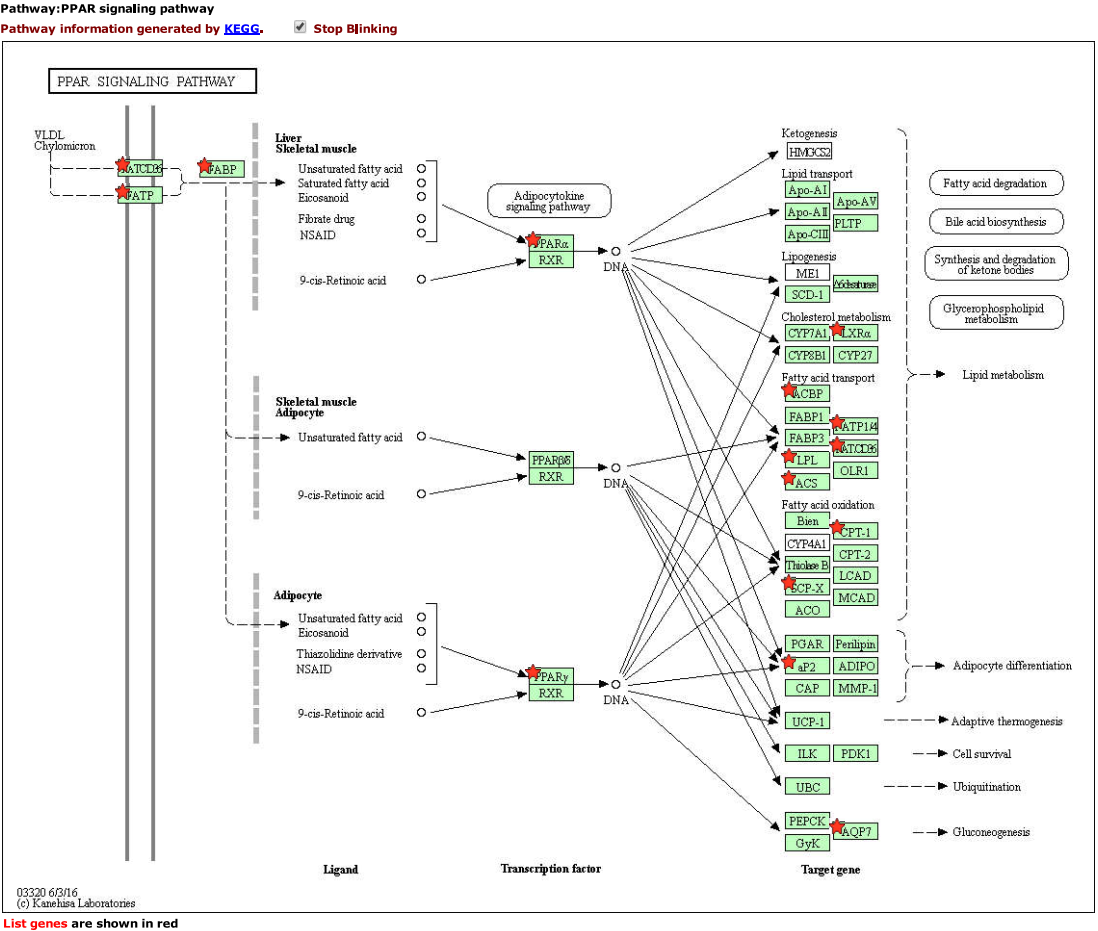


**Figure S1. Different expression genes belonging to PPAR family between diabetic hearts and normal ones.**

Genes altered at 3 day (A), 28 day (B), 42 day (C) after diabetes induction were obtained by statistical reanalysis of the GSE4745 data, and uploaded to online DAVID tools for KEGG enrichment analysis. FA transport and oxidation associated genes were significantly altered in PPAR pathway. Green boxes indicate genes in PPAR pathway. Red stars indicate genes whose expression were significantly changed.

**Figure S2**

**
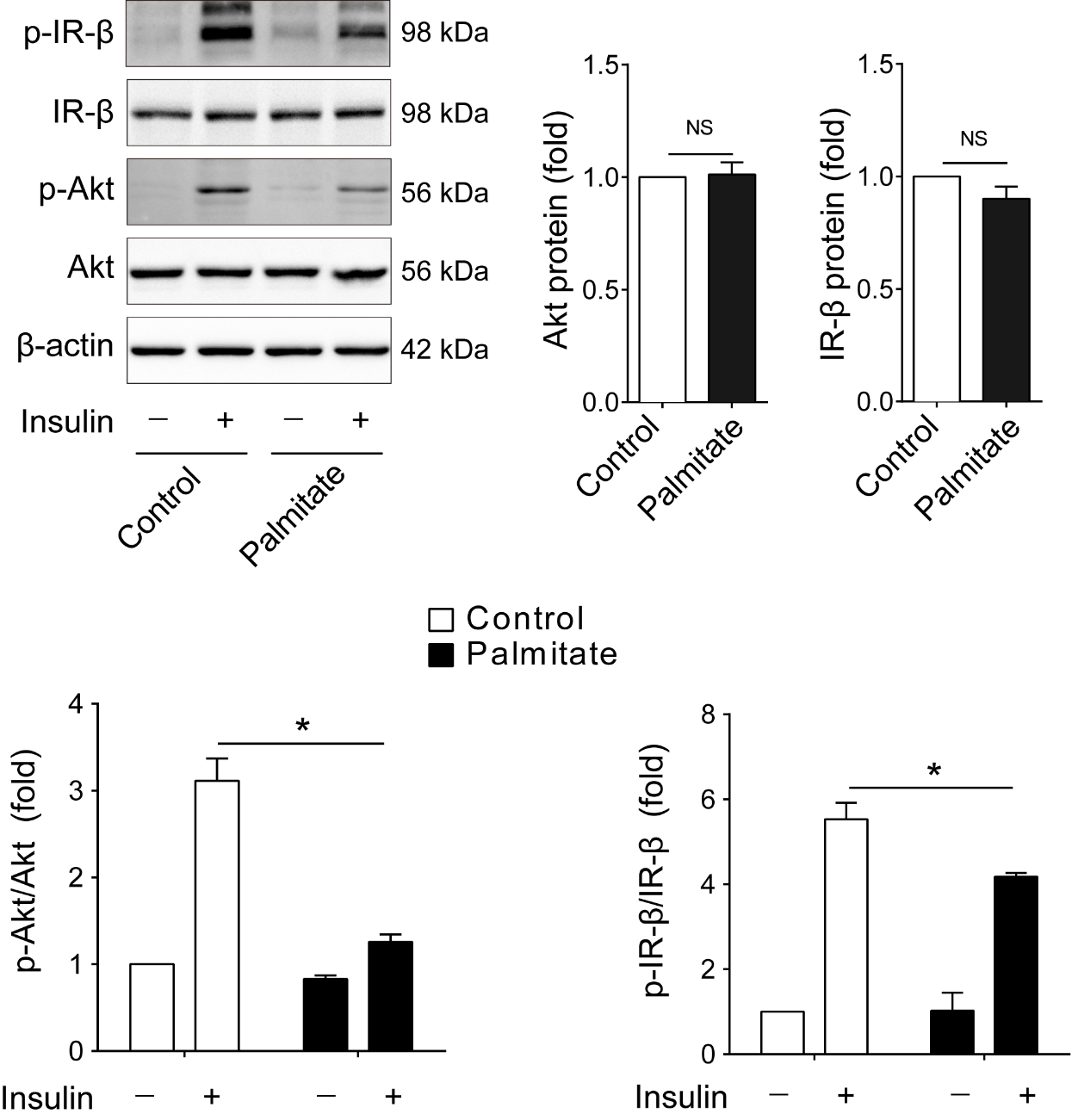
**

**Figure S2. High palmitate treatment impaired the insulin signal of NRCMs.**

NRCMs cells were treated with 300 μM palmitate for 48 hours and then incubated with 10mg/ml insulin 10 min before harvested. Representative Western blots and quantification of IR-β, p-IR-β, Akt and p-Akt in H9c2 cells with different treatment. β-actin was used as an internal control. Data are expressed as mean ± SEM, *p < 0.05.

**Figure S3**


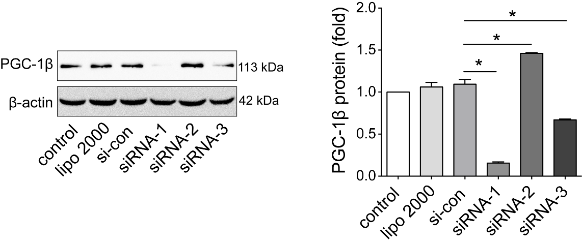


**Figure S3. The efficacy of siRNAs against PGC-1β.**

H9c2 cells were transfected with different PGC-1β siRNA. Representative Western blots and quantification of PGC-1β in H9c2 cells with different treatment. β-actin was used as an internal control. Data are expressed as mean ± SEM, *p < 0.05.

**Figure S4**


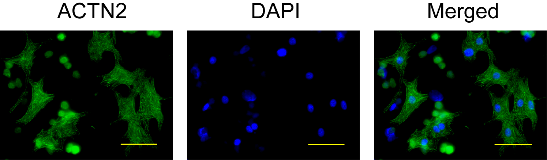


**Figure S4. Identification of the primary** **neonatal rat cardiomyocytes.**

Cells isolated from neonatal rat hearts were stained with antibody against the cardiomyocyte-specific marker α2-actinin (ACTN2) and DAPI. Scale bar=50 μm.

**Figure S5**

**
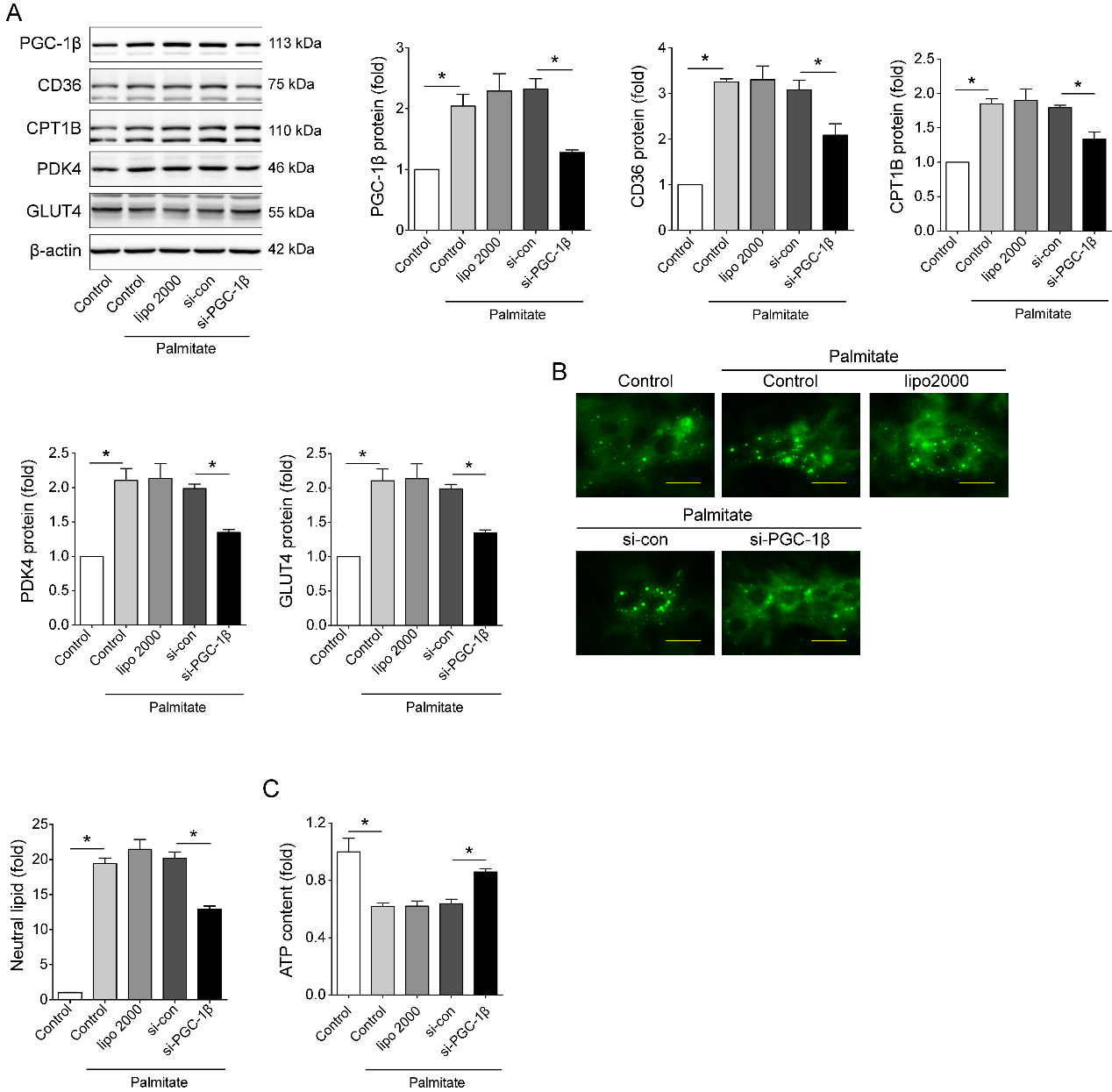
**

**Figure S5. PGC-1β knockdown relieved high palmitate induced lipotoxicity in vitro.**

NRCMs were transfected with PGC-1β siRNA and then subjected to palmitate (300 μM) stimulation. A. Representative Western blots and quantification of PGC-1β, CD36, CPT1B, PDK4, GLUT4 in cells with different treatments. β-actin was used as an internal control. B. Representative images and quantitative analysis of BODIBY 493/503 fluorescent dye staining of neutral lipid level in cells (Bar=25 μm). C. ATP content of H9c2 cells with different treatments. For all panels, data are representative of three independent experiments and expressed as mean ± SEM, *p < 0.05.

**Figure S6**

**
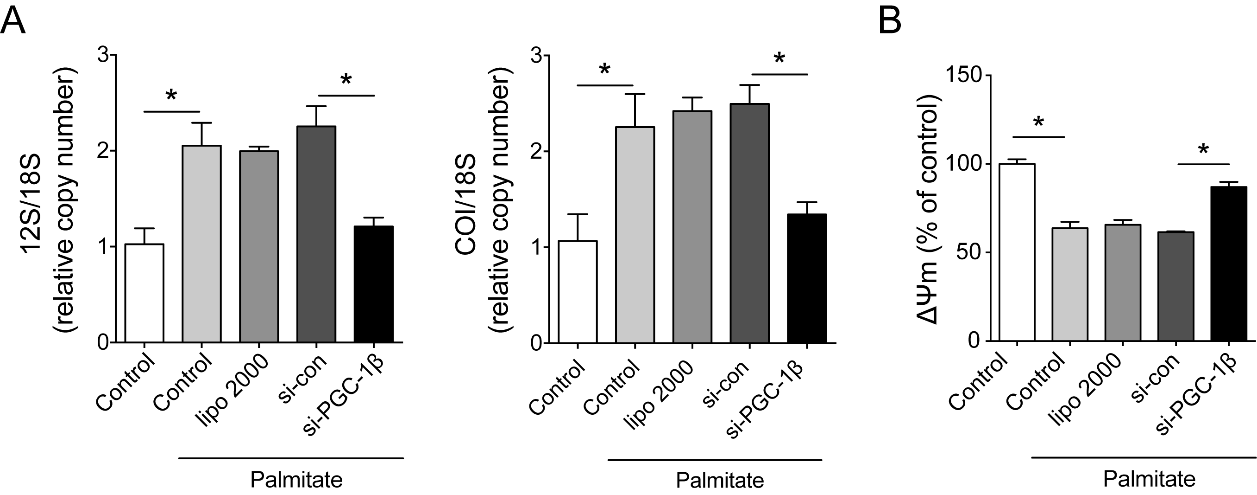
**

**Figure S6. The effects of PGC-1β knockdown on mitochondrial biogenesis and membrane potentials.**

NRCMs were transfected with PGC-1β siRNA and then subjected to palmitate (300 μM) stimulation. A. Graphs show mitochondrial DNA copy numbers relative to genomic DNA that were measured in NRCMs with treatments as indicated using qPCR. B. Mitochondrial membrane potentials (ΔΨm) were analyzed by JC-1 staining in NRCMs with treatments as indicated. For all panels, data are representative of three independent experiments and expressed as mean ± SEM, *p < 0.05.

**Figure S7**

**
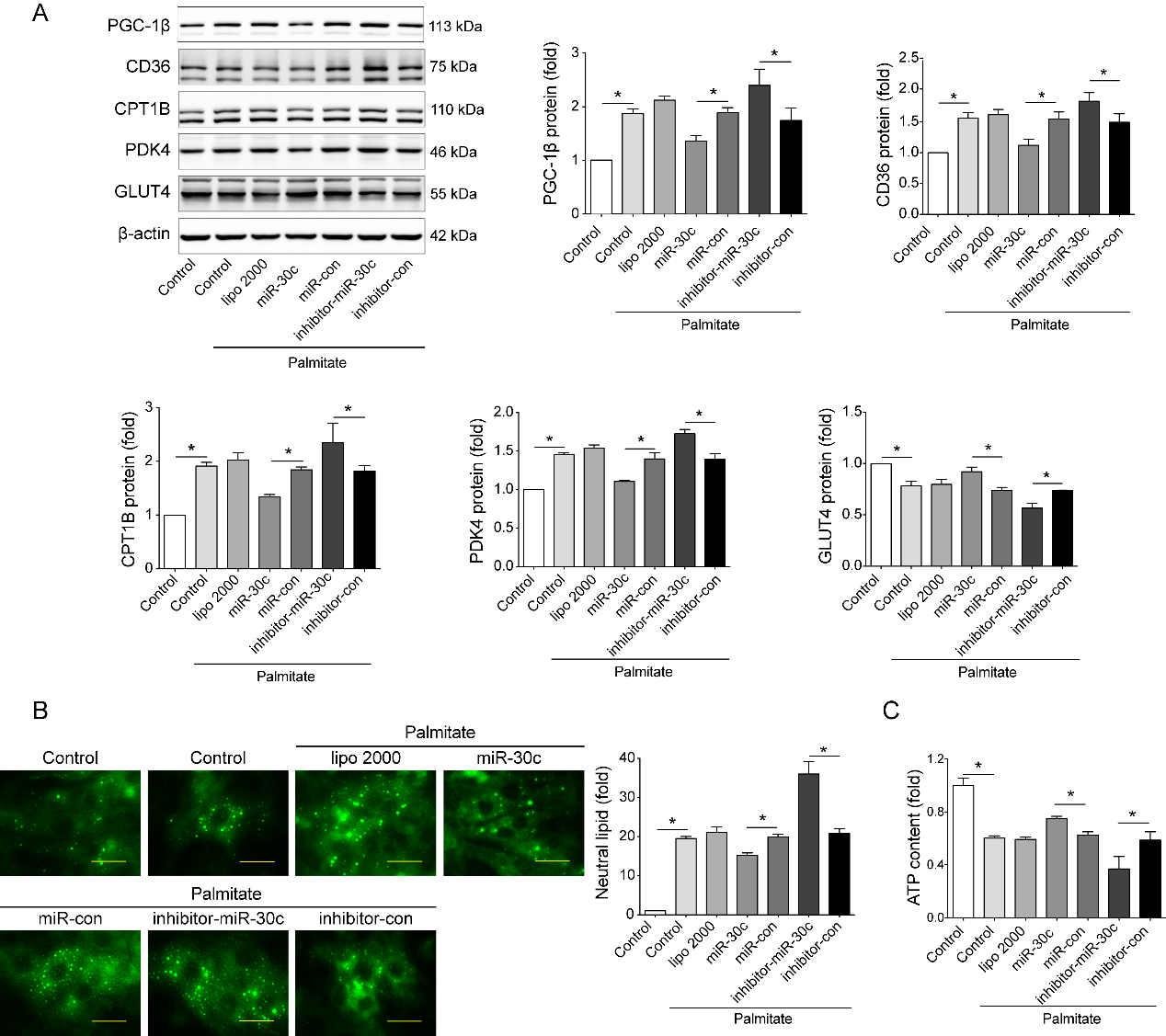
**

**Figure S7. Overexpression of miR-30c alleviated high palmitate induced lipotoxicity in vitro.**

NRCMs were transfected with miR-30c mimics/inhibitors and then subjected to palmitate (300 μM) stimulation. A. Representative Western blots and quantification of PGC-1β, CD36, CPT1B, PDK4, GLUT4 in H9c2 cells with different treatments. β-actin was used as an internal control. B. Representative images and quantitative analysis of BODIBY 493/503 fluorescent dye staining of neutral lipid level in cells (Bar=25 μm). C. ATP content of H9c2 cells with different treatments. For all panels, data are representative of three independent experiments and expressed as mean ± SEM, *p < 0.05.

**Figure S8**


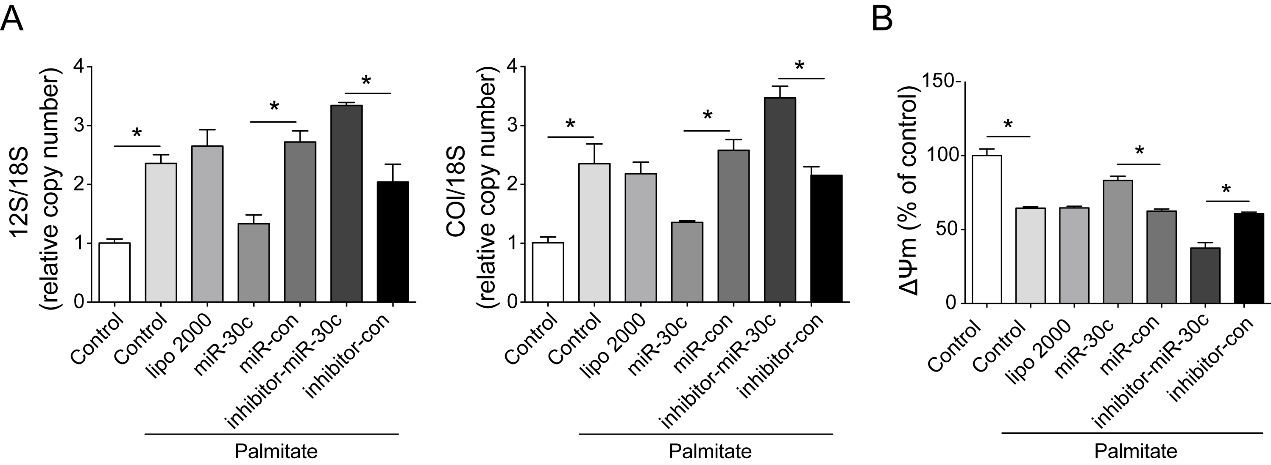


**Figure S8. The effect of miR-30c on mitochondrial biogenesis and membrane potentials.**

NRCMs were transfected with miR-30c mimics/inhibitors and then subjected to palmitate (300 μM) stimulation. A. Graphs show mitochondrial DNA copy numbers relative to genomic DNA that were measured in NRCMs with treatments as indicated using qPCR. B. Mitochondrial membrane potentials (ΔΨm) were analyzed by JC-1 staining in NRCMs with treatments as indicated. For all panels, data are representative of three independent experiments and expressed as mean ± SEM, *p < 0.05.

**Figure S9**

**
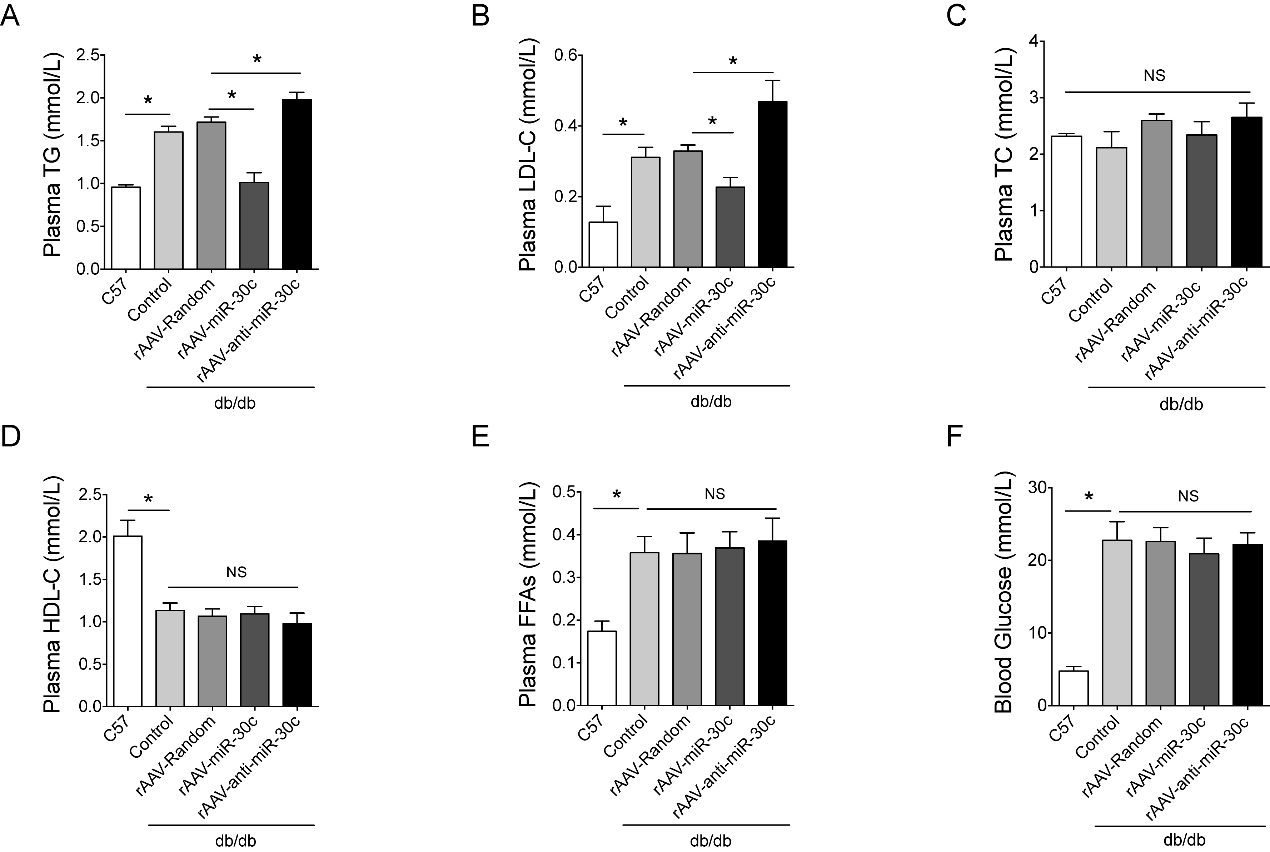
**

**Figure S9. The effects of rAAV9 mediated miR-30c/anti-miR-30c delivery on plasma lipid profile and blood glucose in vivo.**

Db/db mice and C57 control mice were injected with the corresponding rAAVs at 8-10 weeks of age and then sacrificed at 28 weeks of age (n=8-10). The levels of plasma TG (A), LDL-C (B), TC (C), HDL-C (D), FFA (E) and blood glucose (F) in different groups were measured. Data are expressed as mean ± SEM, *p < 0.05. TG, triglycerides; TC, total cholesterol; LDL-C, low-density lipoprotein cholesterol; FFA, free fatty acid.

**Figure S10**

**
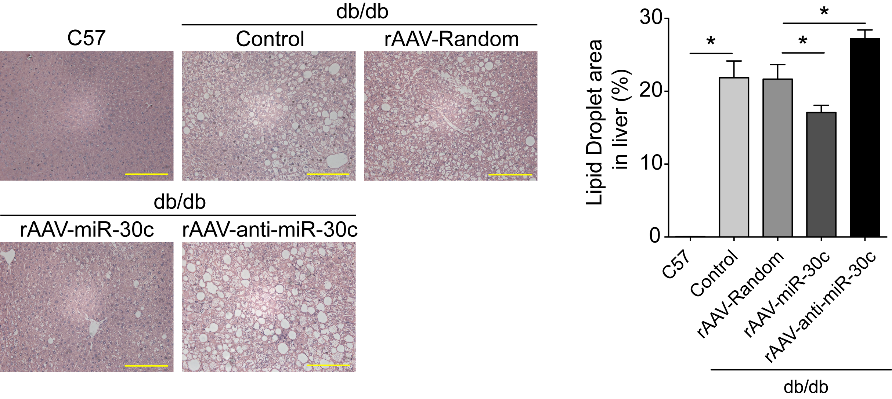
**

**Figure S10. The effects of rAAV9 mediated miR-30c/anti-miR-30c delivery on liver steatosis in vivo.**

Db/db mice and C57 control mice were injected with the corresponding rAAVs at 8-10 weeks of age and then sacrificed at 28 weeks of age (n=8-10). Representative images of HE staining and quantitative analysis of lipid droplets (Bar=200 μm). Data are expressed as mean ± SEM, *p < 0.05.

**Table S1.** The wildtype sequence containing predicted miR-30c binding site of the human PGC-1β 3′ UTR and corresponding mutant sequence.

|  | **Sequences 5′→3′** |
| --- | --- |
| Wildtype | TCGAGGAATACCTCAATACCTCAGACAAGGCCCTTCCAATATGTTTACGTTTTCAAAGAAA |
| mutant | TCGAGGAATACCTCAATACCTCAGACAAGGCCCTTCCAATAAGATAAGGTTTTCAAAGAAA |

**Table S2.** Sequences of miR-30c, anti-miR-30c, or miR-random inserted into rAAV expression plasmid

|  | **Sequence 5′→3′** |
| --- | --- |
| miR-30c | GATCCTGTAAACATCCTACACTCTCAGCTTCAAGAGAGCTGAGAGTGTAGGATGTTTACACCGC |
| anti-miR-30c | GATCCGCTGAGAGTGTAGGATGTTTACATTCAAGAGATGTAAACATCCTACACTCTCAGCCCGC |
| miR-random | GATCCTTTGTACTACACAAAAGTACTGTTCAAGAGACAGTACTTTTGTGTAGTACAAACCGC |

**Table S3.** Primers of CD36 and PDK4 promotors in ChIP-PCR.

|  | **Primer sequence 5′→3′** |
| --- | --- |
| CD36: Forward | GAAATGCCTCAGACCTTCCAGT |
| Reverse | TCAAAACACCTGCTGCCAAG |
| PDK4: Forward | TAAGAAGCAGATGATTGGCTACTGT |
| Reverse | GATTGGCACCCTTGGGATAG |

**Table S4.** Primers of rat 12S ribosomal DNA, COI and and 18S ribosomal DNA in real-time PCR.

|  | **Primer sequence 5′→3′** |
| --- | --- |
| 12S: Forward | GTTATACCTTACCCCTTCTCGC |
| Reverse | CCCATTTCTTTCCGCTTCA |
| COI: Forward | GCTCGTAAACCGTTGACTCT |
| Reverse | TCAGTTCCCGAAGCCTCC |
| 12S: Forward | ACTGGGATTAGATACCCCACTATG |
| Reverse | ATCGATTATAGAACAGGCTCCTC |

**Table S5.** Comparison of hemodynamic variables among mice with different treatments.

|  | C57 | db/db | | | |
| --- | --- | --- | --- | --- | --- |
|  |  | Control | rAAV-Random | rAAV-miR-30c | rAAV-anti-miR-30c |
| HR (bpm.) | 437±19 | 435±19 | 437±26 | 405±12 | 419±12 |
| LVEDP (mmHg) | 1.4±0.6 | 7.6±0.4^*^ | 7.9±0.3 | 4.9±0.5^#^ | 10.2±1.1^#^ |
| LVP_min_ (mmHg) | 1.1±0.2 | 5.7±0.6^*^ | 6.4±0.6 | 4.2±0.2^#^ | 9.7±0.6^#^ |
| LVP_max_(mmHg) | 107.8±1.9 | 98.2±2.5 | 99.9±3.8 | 102.0±1.9 | 90.5±4.1 |

Data are expressed as mean ± SEM, *p < 0.05 vs C57; #p< 0.05 vs rAAV-Random. HR, heart rate; LVEDP, Left ventricular end-diastolic pressure; LVPmin, Left ventricular minimal pressure; LVPmax, Left ventricular maximal pressure.
